# Dietary Patterns and Diet Quality before and/or during Pregnancy and How These Affect Birth Outcomes: A Systematic Review and Meta-analysis

**DOI:** 10.1016/j.advnut.2025.100490

**Published:** 2025-08-12

**Authors:** Cristal Salatas, Anja Bronnert, Robyn Lawrence, Tanith Alexander, Clare Wall, Frank H Bloomfield, Luling Lin

**Affiliations:** 1 Liggins Institute, University of Auckland, Auckland, New Zealand; 2 School of Sport, Exercise and Nutrition, College of Health, Massey University, Auckland, New Zealand; 3 Neonatal Unit, Kidz First, Middlemore Hospital, Auckland, New Zealand; 4 Department of Nutrition, Faculty of Medical and Health Sciences, University of Auckland, Auckland, New Zealand

**Keywords:** preterm birth, low birthweight, diet quality, dietary patterns, pregnancy

## Abstract

Limited consistent evidence exists on how diet quality before and during pregnancy influences preterm birth and low birthweight risk. This study aims to assess whether diet quality based on dietary patterns before and during pregnancy affects preterm birth and low birthweight risk. We systematically searched 3 electronic databases and 4 registries for randomized controlled trials (RCTs) and quasi-RCTs without restrictions on publication date or language until 22 November, 2024. Included RCTs evaluated dietary patterns to enhance diet quality before/during pregnancy compared with a usual diet or placebo. Results were synthesized using random-effects meta-analyses with risk ratios (RRs) and 95% confidence intervals. Study quality was assessed using the Cochrane Risk of Bias 1 tool, and certainty of evidence was evaluated with the Grading of Recommendations Assessment, Development and Evaluation approach. Twenty-nine RCTs (7367 participants) were included. Improved diet quality through dietary patterns providing the recommended macronutrient intake or high unsaturated fats before and during pregnancy reduced the incidence of low birthweight (<2500 g) (7 RCTs, 2178 participants, RR 0.53 [0.37, 0.77], low certainty of evidence) and have potential benefit for reducing preterm birth (15 RCTs, 4949 participants, RR 0.79 [0.62, 1.02], low certainty of evidence) compared with usual diet. The data available support interventions starting in the first trimester (RR 0.30 [0.11, 0.80]), lasting 4–7 mo (RR 0.52 [0.37, 0.73]), with similar effects in both high-/upper-middle-income [RR 0.44 (0.19, 10.04)] and lower-middle-income (RR 0.44 [0.31, 0.63]) populations, especially in low-risk women (RR 0.52 [0.37, 0.73]). Diets providing the recommended macronutrient intake or high in unsaturated fats significantly reduced risk of low birthweight when initiated in the first trimester and maintained for 4–7 mo, regardless of country-level socioeconomic context. Healthcare providers should consider recommending dietary patterns emphasizing whole foods and high-quality fats as part of early prenatal care.

This trial was registered at PROSPERO as CRD42023462517.

**Statement of significance**

Our review provides evidence of the potential importance of initiating dietary interventions early in pregnancy, particularly those providing recommended macronutrient intake or high in unsaturated fat diets in the first trimester, in reducing risk of preterm birth and low birthweight across different socioeconomic settings. Additionally, by exploring how baseline risk status potentially modifies intervention effectiveness, this review offers a more comprehensive understanding of the contextual factors influencing birth outcomes.

## Introduction

Preterm births, defined as infants born before 37 completed weeks of pregnancy, remain a significant global burden, with a global prevalence of 9.9% in 2020 [1]. Complications due to preterm birth are the leading cause of death in children aged under 5 y [1,2]. Many preterm infants who survive experience long-term health problems and neurodevelopmental impairments later in life [1,2]. It is common for preterm infants to be of low birthweight [1], defined as a birthweight <2500 g [3].

Dietary deficiencies or excesses in pregnancy can negatively affect the pregnant woman and the infant during pregnancy and birth [[4], [5], [6]]. Low diet quality during periconception has been associated with adverse birth outcomes such as preterm birth and low birthweight [5,7]. Dietary interventions and adherence to recommended dietary patterns may be a cost-effective and modifiable method of preventing preterm birth and low birthweight.

Investigating if and how dietary patterns and diet quality before and during pregnancy are associated with adverse birth outcomes is crucial for promoting healthy pregnancies. Studies have not found significant protective effects of improved diet quality through specific dietary patterns during pregnancy for reducing risk of preterm birth, despite the diversity in dietary approaches and study populations [[8], [9], [10]]. Further confirmation on the type, duration, timing, and effectiveness of the dietary interventions, especially in a population with varying risk and country-level socioeconomic status, is needed. Therefore, we conducted a systematic review and meta-analyses of randomized trials to assess the associations between diet quality based on dietary patterns as interventions before and during pregnancy and the incidence of adverse birth outcomes.

## Methods

### Protocol and registration

This review was registered prospectively in PROSPERO (http://www.crd.york.ac.uk/PROSPERO; registration number CRD42023462517) and is in accordance with the PRISMA guidelines [11].

### Search strategy

We conducted a comprehensive systematic search for relevant articles from electronic databases, including Ovid MEDLINE, Embase, CINAHL Complete, and the Cochrane Central Register of Controlled Trials from inception to 17 September, 2023, and updated the search up to 22 November, 2024. We also searched for registered trials in Current Controlled Trials (www.controlled-trials.com), Clinical Trials [12], Australian and New Zealand Clinical Trials Registry [13], and WHO International Clinical Trials Registry Platform Search Portal [14]. Conference abstracts were included if they provided useful and robust summary data that we could use. Authors of conference abstracts without corresponding publications were contacted for more information. There were no restrictions on the date of publication or language. The search strategy combined terms related to diet quality based on dietary pattern interventions, pregnancy, and birth outcomes. The complete search strategy is provided in Supplemental Table 1.

### Inclusion criteria

Eligible studies included published RCTs or quasi-RCTs involving pregnant women or those planning pregnancy, with no age restrictions. Interventions focused on any specific dietary patterns, diet quality standards, or modulating dietary patterns and/or diet quality to prevent preterm birth and/or low birthweight at any time before or during pregnancy. Eligible studies reported on the incidence of preterm birth and/or low birthweight. Eligible comparators (control groups) were a placebo treatment, standard/usual care (study-defined), or usual diet.

### Definition of dietary patterns

Dietary patterns included, but were not limited to, the following: the dietary approaches to stop hypertension (DASH) diet, which emphasizes the consumption of fruits, vegetables, whole grains, and low-fat dairy products; the Mediterranean diet, which emphasizes the consumption of whole grains, fruits, vegetables, fish/seafood, beans, and nuts; the new Nordic diet, which replaces processed foods with whole, single-ingredient foods; the paleo diet, which consists of fruits, vegetables, lean meats, fish, eggs, nuts, and seeds; the ketogenic diet, which consists of high-fat, moderate-protein, and very low carbohydrates, and a diet promoting the recommended macronutrient intake (including balanced plate nutrition diet), which consists of carbohydrates 45%–65%, fats 20%–30%, and proteins 15%–25% of total energy [[15], [16], [17], [18]].

### Exclusion criteria

We excluded any study: *1*) that did not report on the outcomes of interest; *2*) in which interventions were a combination of dietary interventions with other interventions (e.g., diet and physical exercise), and *3*) that examined micronutrient supplementation as the only intervention instead of dietary patterns and/or diet quality.

### Outcomes

The primary outcomes were: *1*) preterm birth, defined as a birth before 37 completed weeks of pregnancy, and *2*) low birthweight, defined as infants born weighing <2500 g. Secondary outcomes included: *1*) severity of preterm birth (late preterm, born between 34^+0^ and <37 wk of pregnancy; moderately preterm, born between 32^+0^ and <34 wk of pregnancy; very preterm, born between 28^+0^ and <32 wk of pregnancy; extremely preterm, born before 28^+0^ wk of pregnancy); *2*) the severity of low birthweight (low birthweight, <2500 g; very low birthweight, <1500 g; extremely low birthweight: <1000 g); *3*) mean gestational age (in weeks); and *4*) mean birthweight (in grams).

### Data extraction and quality assessment

Following the study search process, 2 independent authors (CS and AB) screened titles, abstracts, and full texts against predetermined inclusion criteria, and extracted the required data into a data-extraction form using Covidence [19] based on the Population, Intervention, Comparison, and Outcome framework. Data were extracted on the primary and secondary outcomes [preterm birth (and severity of preterm birth) occurrences in the intervention and control groups, low birthweight (and severity of low birthweight) occurrences in the intervention and control groups, gestational age mean and SD, birthweight mean and SD; conducted necessary data transformations if not given mean (SD), otherwise exclude if missing], study characteristics (author, year, location and setting, design, criteria, funding, conflicts, ethics, registration), participant baseline characteristics, group sizes, intervention(s) and comparator(s) details (type, content, timing, duration), and data for determining the risk of bias. A colleague undertook language translation, using DeepL Translate, where necessary.

Two review authors (CS and AB) independently assessed risk of bias for each included study using the Risk of Bias Tool 1 (RoB 1), following the *Cochrane Handbook for Systematic Reviews of Interventions* [20,21]. RoB 1 evaluates studies across 5 domains: sequence generation, allocation concealment, blinding (participants and personnel; outcome assessors), incomplete outcome data, selective reporting, and other sources of bias, assigning ratings of “high risk,” “unclear risk,” or “low risk” for each domain [20]. It does not provide an overall risk of bias judgment. Studies with a low risk of bias across all domains, or unclear risk of bias in 1 domain, were classified as having a low risk of bias. If ≥1 domain was rated as high, the study’s overall quality was considered high. Any disagreements were resolved through consensus or consultation with a third author (LL or RL).

### Data synthesis and statistical analysis

We conducted meta-analyses for each outcome utilizing RevMan 5.4.1 [22], employing the Mantel-Haenszel method with a random-effects model to account for both within- and between-study variability, providing a more generalized estimate of the interventions by assuming that the true effect of an intervention varies across studies rather than being a single fixed value [20,21]. We analyzed the effect size of dichotomous data (preterm birth, low birthweight, severity of preterm birth, severity of low birthweight) as risk ratios (RR) with 95% confidence intervals (CI) to measure the association between dietary patterns and the primary and secondary outcomes. We analyzed the effect size of continuous outcomes (mean birthweight and mean gestational age) as mean differences (MD) with 95% CIs, with the assumption that the outcomes have a normal distribution in the intervention groups of each RCT. Standardized MDs (SMD) were used in outcomes where we assumed that the data collection techniques varied across the included RCTs, with comparable results (different methods to determine gestational age). These estimates were derived directly from raw event counts reported in each trial (intervention compared with control).

A *P* value <.05 signified statistical significance for the outcomes of interest. Data from RCTs reporting quartiles were transformed to obtain means and SDs [23]. Statistical heterogeneity was evaluated using the inconsistency index (*I*^2^ statistic) and χ^2^ test. An *I*^2^ > 50% and *P* < .10 in the χ^2^ test were considered to indicate significant heterogeneity. If there was evidence of clinical heterogeneity sufficient to expect that the underlying treatment effects differed between RCTs, or if substantial statistical heterogeneity was detected, we performed subgroup analyses to explore this heterogeneity for the primary outcomes (preterm birth and low birthweight). If numbers permitted, sensitivity analysis was conducted by examining only those RCTs with a low risk of bias for all 5 bias domains. We assessed publication bias by inspecting the funnel plot when >10 RCTs were available for the analysis. If asymmetry was apparent, we considered and discussed possible reasons for it.

We performed prespecified subgroup analyses to evaluate differences in the primary outcomes (preterm birth and low birthweight) based on the duration of the intervention before and during pregnancy [<1 mo, 1–3 mo, 4–7 mo, 8–9 mo, >9 mo (for RCTs reporting only trimester of intervention, duration was estimated by the difference in the starting and ending trimesters)] and the baseline risk of a preterm birth and/or low birthweight infant (high risk: due to previous preterm birth or low birthweight delivery, metabolic risks, gestational diabetes, obesity/overweight, participants receiving in vitro fertilization, or study defined; and low/no risk). We performed additional nonprespecified subgroup analyses to investigate the effect of the timing of dietary intervention (preconception, first, second, or third trimester, and across trimester/no specific trimester), dietary patterns (Mediterranean/DASH diet, high-fiber diet, low carbohydrate/sugar diet, and other or undefined diets), the macronutrient-specific interventions [low-carbohydrate, high-carbohydrate, high-fat-quality/unsaturated fats, and recommended macronutrient intake diets (DASH diet and balanced plate nutrition diet)], and the study country classifications by income level (high-income, upper-middle-income, lower-middle-income, and low-income) on the primary outcomes (preterm birth and low birthweight).

To assess the robustness of risk estimates of the primary outcomes (preterm birth and low birthweight) and minimize bias from confounding factors such as regional and population differences, we conducted stratified random-effects meta-analyses using the inverse variance method and the Der Simonian-Laird estimator to account for between-study heterogeneity [24]. Geographical regions from the included studies were categorized into 2 broader groups (high/upper-middle-income and low/lower-middle-income) based on World Bank classifications [25]. This stratified analysis was exploratory in nature and focused specifically on dietary patterns that demonstrated statistically significant associations with the outcomes in the subgroup analyses assessing the type of macronutrient-specific interventions. This approach allowed us to examine whether the observed associations within that subgroup varied by socioeconomic context and helped account for heterogeneity across studies. The characteristics of studies table (Table 1) [[26], [27], [28], [29], [30], [31], [32], [33], [34], [35], [36], [37], [38], [39], [40], [41], [42], [43], [44], [45], [46], [47], [48], [49], [50], [51], [52], [53], [54], [55], [56], [57], [58], [59], [60], [61], [62], [63]] include details of each RCT and helped decide which RCTs were eligible for each prespecified analysis.

**TABLE 1:** Characteristics of included studies (RCTs and cluster-RCTs).

Ref	Country/date	Sample size (*n*)	Participants	Duration of intervention	Intervention	Intervention food items	Comparator	Comparator food items	Outcome and significance
Alamolhoda et al., 2022 [26]	Iran/2014–2016	Intervention: 455 comparator/control: 457	Inclusion criteria: pregnant women who had a gestational age <8 wk, singleton pregnancy and intention to receive prenatal care, prepregnancy BMI 18–24 kg/m^2^, 18–35 y old, gravid <4, abortion ≤2. Exclusion criteria: history of chronic diseases such as diabetes mellitus, any history of preeclampsia or gestational diabetes, any history of prepregnancy hypertension, and those who smoked or drank alcohol.	Before 8 wk of gestation until delivery (4–7 mo)	Individualized dietary patterns with low trans fatty acid (TFA) content	Individualized dietary patterns with TFA content under 1% of total daily energy intake. In the intervention group, we replaced mono- and PUFAs free of trans fatty acids in dietary patterns, such as olive oil, fish, nuts, and low fat (1.5%) dairy products, and participants were forbidden to consume the food rich of trans fatty acids kind of fast foods, processed meat, and deep fried foods. All pregnant women who participated in the study routinely received 1 mg folic acid daily in the first trimester (≤12 wk) and received multivitamin minerals daily from the beginning of the second trimester to the end of pregnancy. From the beginning of the 16th wk of the pregnancy, all participants received the 30 mg daily iron supplement.	Individualized dietary patterns without any focus on TFA content.	Women were allowed to consume any kind of dairy products and oil for cooking and routine dietary recommendation were advised. All pregnant women who participated in the study routinely received 1 mg folic acid daily in the first trimester (≤12 wk) and received multivitamin minerals daily from the beginning of the second trimester to the end of pregnancy. From the beginning of the 16th week of the pregnancy, all participants received the 30 mg daily iron supplement.	Primary: low birthweight (LBW) ↓ Secondary: gestational age (GA) ↔
Allehdan et al., 2022 [27]	Jordan/2017–2019	Intervention: 25 Comparator/control: 26	Inclusion criteria: pregnant Jordanian women aged between 20 and 46 y had gestational diabetes (GD) and in use of metformin with singleton pregnancies between 24th and 30th wk of gestation. Exclusion criteria: women with multiple gestations, personal history of cardiovascular, kidney, liver and autoimmune diseases, type 1 or type 2 diabetes (except history of GDM), and a positive OGTT before 24th wk of pregnancy consistent with diagnosis of overt diabetes in pregnancy. Women who have contraindications for metformin use, major fetal malformation that was recognized on ultrasound examination or preterm rupture of membrane or placenta abruption at study enrolment, as well as those who take medication that influences glucose metabolism, such as continuous therapy with oral corticosteroids were excluded.	24–30 wk of gestation until delivery (1–3 mo)	Intervention group 1: carbohydrate counting diet (CHO) Intervention group 2: carbohydrate counting (CHO) and DASH diet	Intervention group 1: the calorie content and protein composition combined with carbohydrate counting were comparable to the control diet. Intervention group 2: the calorie content and protein composition combined with carbohydrate counting were comparable to the control diet; however, the DASH diet was rich in fruits (3–4 servs/d), vegetables (4–5 servs/d), cereals [at least half of the total servings were whole grain (6–8 servings/d)], low-fat dairy products (2–4 servings/d), low in lean meat (0–2 servings/d), and nuts, seeds, and legumes (4–5 servings/wk). Adequate intake of sodium (<2400 mg/d) was applied into participants’ diet.	Standard diet	45%–55% carbohydrates, 15%–20% protein, 25%–30% total fat. This was distributed into 3 moderate-sized meals and 2–4 snacks.	Primary: preterm birth (PTB) ↔ Secondary: birthweight (BW) ↔ GA ↔
Al Wattar et al., 2019 [28]	England/2014–2016	Intervention: 627 Comparator/control: 625	Inclusion criteria: ≥16 y of age, <18 wk of gestation with a singleton pregnancy, able to consume nuts and olive oil, and proficient in written and spoken English. Exclusion criteria: history of pre-existing diabetes, gestational diabetes, chronic renal disease, or autoimmune disease, or if they were taking lipid-altering drugs such as statins at the time of booking	Before 18 wk of gestation until delivery (4–7 mo)	Mediterranean-style diet	High intake of nuts, extra virgin olive oil, fruit, vegetables, nonrefined grains, and legumes; moderate-to-high consumption of fish; low-to-moderate intake of poultry and dairy products such as yogurt and cheese; low consumption of red meat and processed meat; and avoidance of sugary drinks, fast food, and food rich in animal fat. To promote their intake in pregnancy, we provided participants in the intervention arm with mixed nuts (30 g/d of walnuts, hazelnuts, and almonds) and extra virgin olive oil (0.5 L/wk) as the main sources of cooking fat.	Standard diet	Dietary advice as per UK national recommendations for antenatal care and weight management in pregnancy.	Primary: PTB ↔ Secondary: BW ↔
Asemi et al., 2014 [29]	Iran/2013	Intervention: 29 Comparator/control: 29	Inclusion criteria: primigravida pregnant women aged <40 y diagnosed with GDM by a 100-g oral glucose tolerance test at 24–28 wk of gestation Exclusion criteria: those with a previous glucose intolerance/GDM diagnosis, premature preterm rupture of membrane, placenta abruption, preeclampsia, requiring commencing insulin therapy during intervention, complete bed rest, hypothyroidism, urinary tract infection, smoking and kidney or liver diseases, as well as those taking estrogen therapy.	24–28 wk of gestation for 4 wk (1–3 mo)	DASH diet	45%–55% carbohydrates, 15%–20% protein, 25%–30% total fat, fruits, vegetables, whole grains, and low-fat dairy products, and low in saturated fats, cholesterol, refined grains, and sweets. The amount of sodium intake was 2400 mg/d + 400 mg/d folic acid from the beginning of pregnancy and 50 mg/d ferrous sulfate as well as multivitamin mineral supplements from 20 wk of gestation.	Standard diet	45%–55% carbohydrates, 15%–20% protein, 25%–30% total fat	Primary: Secondary: BW ↓ GA ↔
Assaf-Balut et al., 2017 [30]	Spain/2015	Intervention: 500 Comparator/control: 500	Inclusion criteria: 18 y or older, single gestation, acceptance of participation in the study, and signature of the consent form. Exclusion criteria: gestational age at entry >14 wk, intolerance to nuts or extra virgin olive oil (EVOO), medical conditions or pharmacological therapy that could compromise the effect of the intervention and/or the follow-up program.	First antenatal visit (<14 wk of gestation) until delivery (4–7 mo)	Mediterranean-style diet	Daily consumption of ≥40 mL of EVOO and a handful (25–30 g) of pistachios. To ensure the consumption of the minimum amount recommended, women were provided at visits 1 and 2 with 10 L of EVOO and 2 kg of roasted pistachios each. This way, they had available 1 L of EVOO and 150 g of roasted pistachios weekly, throughout the pregnancy, and fresh juices, fast foods, and precooked meals.	Standard diet	Restrict consumption of dietary fat, including EVOO and nuts. Two servings/d of vegetables, 3 servings/d of fruit (avoiding juices), 3 servings/d of skimmed dairy products, wholegrain cereals, 2–3 servings of legumes/wk, moderate-to-high consumption of fish; a low consumption of red and processed meat, avoidance of refined grains, processed baked goods, presliced bread, soft drinks and fresh juices, fast foods and precooked meals.	Primary: PTB ↓ Secondary: BW ↔ GA ↔
Belfort et al., 2023 [31]	Brazil/2016–2020	Intervention: 40 Comparator/control: 47	Inclusion criteria: chronological age ≥18 y at conception, diagnosis of diabetes mellitus (DM) (types 1 or 2) with onset before pregnancy, single fetus, gestational age ≤28 wk at recruitment, nonsmoker, and nondrinker. If a candidate had chronic systemic arterial hypertension, they were still included, provided it was mild and controlled (systolic blood pressure ≥140 and <160 mmHg and/or diastolic blood pressure ≥90 and ≤110 mmHg) and they were not diagnosed with hypertensive disorders of pregnancy (gestational hypertension, preeclampsia, or eclampsia). Women with treated and controlled hypothyroidism were also included. Exclusion criteria: the study excluded women with DM complications, such as nephropathy, retinopathy, heart or liver diseases, or other chronic or infectious diseases that could alter the course of pregnancy, and those who had eating disorders.	Before 28 week of gestation until delivery for 18 wk (4–7 mo)	DASH diet	Carbohydrates accounted for 45%–55% of total energy intake (TEI), protein for 15%–20% and lipids for 25%–30%. The use of sucrose was not recommended, but if the woman had chosen to consume food with sucrose, its energy value was computed in the TEI. Each dietary plan was divided into 5–6 meals a day at regular times, and the energy distribution in each of them was planned for both groups so that the main meals contained a higher proportion of energy (lunch and dinner – 20%–30% TEI) and the smaller meals contained a lower proportion (breakfast–10%–15%; mid-morning snack—5%–10%; afternoon snack—10%–15%; supper—5%–10% TEI). Received a portion of seeds (200 g), nuts (150 g), and skimmed milk powder (280 g) on each visit. At their first consultation, all the women received 1 bottle (500 mL) of extravirgin olive oil.	Standard diet	Followed their habitual diet and received a portion of oats (250 g) and a portion of low-fat milk powder (1%–2%, 300 g). At their first consultation, all the women received one bottle (500 mL) of extravirgin olive oil.	Primary: PTB ↔ LBW ↔ Secondary: BW ↔
Chen et al., 2018 [32]	China/2016–2016	Intervention: 31 Comparator/control: GDM control: 31, Health control: 30	Inclusion criteria: age 22–35, singleton, no other underlying diseases, diagnosed with GDM according to the 2016 American Diabetes Association diagnostic criteria at 24–28 wk of pregnancy. Exclusion criteria: not stated	24–28 wk of gestation for 12 wk (1–3 mo)	Low glycemic index diet (LGI)	A natural mix of grains such as black rice, buckwheat, oats, and whole barley in proportions of 100–150 g per day. A specific diet plan was designated, aiming for a total glycemic index (GI) value <55, meeting the requirements of a low-GI diet.	Standard diet	GDM control: general guidance on controlling total energy intake, with a mixed diet having a total glycemic index (GI) >55 Health control: followed their regular diet	Primary: PTB ↔ LBW ↔ Secondary: BW ↔ GA ↔
Chowdhury et al., 2017 [33]	Bangladesh/2016-2017	Intervention: 445 Comparator/control: 448	Inclusion criteria: married and of reproductive age (15–49 y), pregnant with a duration of gestation of 7–12 wk, permanent residents of the study area not expecting to move from the usual residence until birth. Exclusion criteria: women who have planned to deliver outside the study area or have been diagnosed with any chronic diseases, such as diabetes, hypertension, and other diseases that may impact their ability to participate in the trial.	First trimester (7–12 wk of gestation) until delivery (4–7 mo)	Balanced plate nutrition education	(a) Intake of food-yielding ≥2500 kcal energy per day; (b) consumption of diversified food; (c) inclusion of animal sourced food in the diet, ≥2 servings/d; and (d) eating ≥ 5 times a day (5 different plates for 5 meals). This contained 7-food groups (cereal, lentils, animal protein, vegetables, fruits, milk, and oil), giving a daily 2500 kcal energy and essential micro and macronutrients. The balanced plate was a combination of foods in appropriate portion sizes to meet requirements.	Standard diet	Pregnant women received the same frequency of home and standard nutrition advice except for the balanced plate demonstrations. Standard advice recommended consuming all food groups and taking iron-folic acid and calcium supplements.	Primary: LBW ↓ Secondary: BW ↑
Crovetto et al., 2021 [34]	Spain/2017–2020	Intervention: 407 Comparator/control: 407	Inclusion criteria: 18 y or older, fluency in Spanish language, singleton pregnancy, positive fetal heart rate at the time of ultrasonography, and high risk of newborns with small for gestational age (SGA) according to the adapted criteria of the Royal College of Obstetricians and Gynaecologists for SGA. Exclusion criteria: fetal anomalies, including chromosomal abnormalities, structural malformations, and congenital infections, detected prenatally; neonatal malformations or congenital anomalies diagnosed after birth; inability to perform additional visits; participation in another trial; and maternal intellectual disability or other mental or major psychiatric disorders requiring therapy during pregnancy.	21 wk of gestation until delivery (4–7 mo)	Mediterranean-style diet	All participants received olive oil (2 L every month) and 15 g of walnuts per day (450 g every month). The participant received dietary training and personalized advice to increase adherence to the Mediterranean diet, including recipes, a quantitative 1-wk shopping list of food items according to the season of the year, and a weekly plan of meals with detailed menus that were also available on the trial website. Participants were encouraged to increase the intake of whole grain cereals (5 servings/d); vegetables and dairy products (3 servings/d); fresh fruit (2 servings/d); and legumes, nuts, fish, and white meat (3 servings/wk), as well as olive oil use for cooking and dressing.	Standard diet	Standard prenatal care	Primary: PTB ↔ Secondary:
Facchinetti et al., 2019 [35]	Italy/not stated	Intervention: 139 Comparator/control: 134	Inclusion criteria: Italian women between the ninth and 12th wk of gestation with BMI≥ 25 m/kg^2^, aged >18 y, and with a singleton pregnancy. Exclusion criteria: chronic diseases, which included diabetes mellitus (first-trimester glycosuria >100 mg/dL or fasting plasma glucose≥ 126 mg/dL, which are part of the routine tests prescribed in antenatal clinics, or random glycaemia≥ 200 mg/dL), chronic hypertension, other medical conditions or dietary supplementations that might affect body weight (i.e., untreated thyroid diseases), previous bariatric surgery, smoking habits, and contraindications to practicing physical activity.	9–12 wk of gestation until delivery (4–7 mo)	Low glycemic index diet (LGI)/low-carb diet	Caloric restriction consisted of the prescription of a low-GI, low-saturated fat diet with a total intake of 1500 kcal/d. 200 kcal/d for obese and 300 kcal/d for overweight women were added. The prescribed diet was based on the wide consumption of plant foods, cereals, legumes, and fish, with olive oil as the main source of fat, and moderate-to-no consumption of beer, wine, and other alcoholic beverages. The dietary plan provided 3 main meals and 3 snacks (breakfast, snack, lunch, snack, dinner, and evening snack before bedtime). For each 1 of the meals or snacks, the pregnant women had several alternatives, all of which were calibrated suitably. Food alternatives were offered and chosen according to individual choices.	Standard diet	All of the women received a general nutritional booklet regarding correct lifestyle, namely about a proper food intake without a specific caloric restriction, in accordance with the Italian guidelines.	Primary: Secondary: BW ↓
Geiker et al., 2022 [36]	Denmark 2014–2017	Intervention: 138 Comparator/control: 141	Inclusion criteria: women aged older than 18 y, with a prepregnancy BMI of 28–45 kg/m^2^ and a singleton pregnancy. Exclusion criteria: women unwilling or unable to provide informed consent; had experienced fluctuations in weight >10 kg over the last year; had underlying disorders that could interfere with the intervention; abused alcohol or drugs; and had allergy or intolerance to dairy or fish.	Before 15 wk of gestation until delivery (4–7 mo)	High-protein low glycemic index (HPLGI)	High in protein (25%–28% of total energy consumed) and low in glycemic index (maximum mean of 55 glycemic index units from the total diet), weighted by the carbohydrate content of the individual food items. Moderate fat content (30% of total energy) and the women were instructed not to restrict their energy consumption, i.e., to consume the diets ad libitum, to evaluate potential appetite- and weight-regulating effects. All women were provided with daily supplements of fish oil capsules containing vitamin B-12 and folate, vitamin D, and iron.	Moderate-protein moderate glycemic index (MPMGI)	Followed the Nordic Nutritional Recommendations and included a moderate-protein content (18% of total energy consumed) and no instructions with respect to the glycemic index, i.e., a moderate glycemic index. Moderate fat content (30% of total energy) and the women were instructed not to restrict their energy consumption, i.e., to consume the diets ad libitum, to evaluate potential appetite- and weight-regulating effects. All women were provided with daily supplements of fish oil capsules containing vitamin B-12 and folate, vitamin D, and iron.	Primary: PTB ↔ LBW ↔ Secondary: GA ↑ BW ↔
Grant et al., 2011 [37]	Canada 2006–2007	Intervention: 10 Comparator/control: 16	Inclusion criteria: pregnant women aged 18–45 y, diagnosed with gestational hyperglycaemia, defined as gestational diabetes mellitus (GDM) or impaired glucose tolerance of pregnancy, and who had been referred to the Diabetes in Pregnancy Clinic (DIP), St. Michaels Hospital were eligible to participate. Exclusion criteria: presence of a multiple pregnancy or an acute or chronic illness affecting carbohydrate metabolism; presence of type 1 or type 2 diabetes before the current pregnancy; use of insulin treatment before providing consent; >34 wk of gestation; and unable to communicate in English with no translator available.	29 wk of gestation until delivery (4–7 mo)	Low glycemic index diet (LGI)	Participants randomly assigned to the low-GI group were given a list composed of low-GI foods. Women enrolled in our study were asked to select their starch choices from an exchange list specific to their assigned study group.	Standard diet	The control group was given a list of intermediate- and high-GI foods; reflecting the usual intake of typical DIP Clinic patients.	Primary: Secondary: BW ↓
Hernandez et al., 2023 [38]	United States/2015–2020	Intervention: 31 Comparator/control: 28	Inclusion criteria: English-speaking pregnant women with singleton pregnancies, who were between 20 and 39 y old, and had BMI of 26–42 kg/m^2^ at the time of diagnosis (expanded from the original BMI range of 26–39 kg/m^2^ after 1 y) allowed greater participation in this study. Women with GDM meeting criteria by a 50-g glucose challenge followed by a 3-h 100-g oral glucose tolerance test (OGTT) according to the American College of Obstetricians and Gynecologists, supported by the American Diabetes Association and not requiring medication. Exclusion criteria: women with fasting triglyceride level ≥400 mg/dL, who smoked, had pre-existing diabetes, or had suspected pre-existing diabetes (A1C ≥6.5%, fasting glucose level >125 mg/dL, or random glucose level >200 mg/dL) or women un-likely to achieve glycemic control with diet alone (fasting glucose level >105 mg/dL) were not enrolled. Women with risk factors for placental insufficiency, including hypertension, renal disease, thrombophilia, rheumatologic disease, a history of pre-eclampsia, or fetal growth restriction were excluded, as were those with a history of preterm labor, and those who used b-blockers or glucocorticoids. Women requiring insulin, oral hypoglycemic agents, or delivery at <36 wk of gestation were removed from the study.	30–31 wk of gestation until delivery for 7–8 wk (1–3 mo)	High carbohydrate, low fat [CHOICE diet (Choosing Healthy Options in Carbohydrate Energy)]	60% complex carbohydrates, 25% fat, and 15% protein, consistent with the American Heart Association guideline to reduce cardiovascular disease risk. Protein, fat, and sugars in both diets were as follows: 15% protein provided ≥71 g/d, which met Institute of Medicine (IOM) guidelines; daily fat was 35% SFA, 45% MUFA, and 20% PUFA; and simple sugars were fixed at 70 ± 5 g/d (absolute grams) in both diets. Daily calories were partitioned as 25% (breakfast), 25% (lunch), and 30% (dinner), and the remaining 20% was divided into snacks. Both diets consisted of mainly low (≤55) to moderate (56–69) glycemic index foods.	Conventional lower-carbohydrate (40%)/higher fat (45%) diet (LC/CONV)	40% carbohydrate, 45% fat, and 15% protein. Protein, fat, and sugars in both diets were as follows: 15% protein provided ≥71 g/d, which met Institute of Medicine (IOM) guidelines; daily fat was 35% SFA, 45% MUFA, and 20% PUFA; and simple sugars were fixed at 70 ± 5 g/d (absolute grams) in both diets. Daily calories were partitioned as 25% (breakfast), 25% (lunch), and 30% (dinner), and the remaining 20% was divided into snacks. Both diets consisted of mainly low (≤55) to moderate (56–69) glycemic index foods.	Primary: Secondary: GA ↔ BW ↔
Jiang et al., 2019 [39]	China/2015–2017	Intervention: 52 Comparator/control: 47	Inclusion criteria: age between 20 and 40 y, <28 wk of gestation at time of the first clinical visit, physician-diagnosed GH and/or chronic hypertension, and providing their consent to take part in the project. Exclusion criteria: inability to consent, medical or surgical diseases (i.e., a specific psychiatric disorder, immunological, liver and kidney diseases), smoking, alcohol abuse and long-term medication (i.e., psychotropic, anti-hyperlipidemic and immunosuppressive drugs).	Less than 28 wk of gestation until delivery for 12 wk (1–3 mo)	DASH diet	Modified diet to accommodate specific needs of pregnancies and was similar to the control diet in terms of macronutrients (50%–60% carbohydrate, 20%–25% fat and 20%–25% protein); however, it was rich in fruits, vegetables, whole grains and low-fat dairy products, as well as low in saturated fats, cholesterol and sweets. The amount of salt intake was 4 g day 1.	Standard diet	Consumed a medical nutrition therapy diet, as a percentage of total energy [ideal body weight 9 (25–35) kcal day 1], a macronutrient distribution of 50%–60% carbohydrate, 25%–30% fat and 15%–20% protein. All pregnant women consumed supplements (i.e., calcium and folic acid) and were provided with anticoagulant drugs (i.e., aspirin) as needed.	Primary: PTB ↓ LBW ↓ Secondary: GA ↑ BW ↔
Louie et al., 2011 [40]	Australia/2008–2010	Intervention: 47 Comparator/control: 45	Inclusion criteria: women aged 18–45 y diagnosed with GDM by a 75-g oral glucose tolerance test at 20–32 wk of gestation, with an otherwise healthy singleton pregnancy, were eligible for the study. Exclusion criteria: women who had special dietary requirements (including vegetarianism/veganism), pre-existing diabetes, or pregnancy achieved by assisted reproduction and those who smoked or consumed alcohol during pregnancy were excluded.	29 wk of gestation until delivery (1–3 mo)	Low glycemic index diet (LGI)	Similar protein (15%–25%), fat (25%–30%), and carbohydrate (40%–45%) content with an LGI (target GI ∼50).	HF (high-fiber content and moderate-GI)	Similar protein (15%–25%), fat (25%–30%), and carbohydrate (40%–45%) content with a high-fiber content and moderate-GI, similar to the Australian population average (HF) (target GI ∼60).	Primary: PTB ↔ Secondary: GA ↓ BW ↔
Markovic et al., 2016 [41]	Australia/2011–2012	Intervention: 76 Comparator/control: 71	Inclusion criteria: women aged >18 y between 12 and 20 wk of gestation and at high risk of GDM with an otherwise healthy single pregnancy. Women were considered to be at high risk if they had ≥1of the following risk factors: age >35 y, first-degree relative with T2DM, prepregnancy BMI 30 kg/m^2^, history of GDM or glucose intolerance, history of a previous infant >4000 g, or belonging to a high-risk ethnic group (Aboriginal or Torres Strait Islander, Polynesian, Middle Eastern, Indian, or Asian). Exclusion criteria: pre-existing diabetes or special dietary requirements (including vegetarianism/veganism.	17 wk of gestation until delivery (4–7 mo)	Low glycemic index diet (LGI)	Protein (15%–25% total energy intake), fat (25%–30%), and carbohydrate content (40%–45%). Target GI ∼50.	High-fiber, moderate-GI diet (HF)	Protein (15%–25% total energy intake), fat (25%–30%), and carbohydrate content (40%–45%). Target GI similar to the Australian population average (target GI ∼60).	Primary: PTB ↔ LBW ↔ Secondary: GA ↔ BW ↔
Mijatovic et al.,2020 [42]	Australia/2016–2018	Intervention: 24 Comparator/control: 22	Inclusion criteria: pregnant women aged 18–45 y with a singleton pregnancy, between 24 and 32 wk of gestation with a GDM diagnosis. Exclusion criteria: women who consumed alcohol, smoked cigarettes, followed a gluten-free, vegetarian, or vegan diet, had assisted reproduction, could not understand English, had major surgery (e.g., gastric bypass) in the previous 5 y, or had comorbidities other than obesity, hypertension, or dyslipidemia.	24–32 wk of gestation for 6 wk (1–3 mo)	Modestly lower carbohydrate diet	Absolute carbohydrate target of 135 g/d without energy restriction, based on the estimated average requirement for carbohydrate intake during pregnancy.	Standard diet	Aimed for an absolute carbohydrate target of 180–200 g/d.	Primary: Secondary: GA ↔ BW ↓
Moreno-Castilla et al.,2013 [43]	Spain/2008–2011	Intervention: 75 Comparator/control: 75	Inclusion criteria: women aged 18–45 y (inclusive) diagnosed with GDM with singleton pregnancies and a gestational age ≤35 wk. Exclusion criteria: unwillingness to follow a prescribed diet, inability to understand the Spanish language, and pregnancy comorbidities other than obesity, hypertension, and/or dyslipidemia.	30 wk of gestation until delivery (1–3 mo)	Low-carbohydrate diet (CHO)	Low content of CHO (40% of calories) divided into 3 principal meals and 3 snacks, all with a prespecified number of CHO servings.	Control diet (average carbohydrate content)	Control diet (55% of calories) divided into 3 principal meals and 3 snacks, all with a prespecified number of CHO servings.	Primary: Secondary: GA ↔
Moses et al., 2006 [44]	Australia/not stated	Intervention: 32 Comparator/control: 30	Inclusion criteria: healthy pregnant women aged 21–40 y, had a singleton pregnancy, were between 12 and 16 wk of gestation, were nonsmokers, and had no >1 alcoholic drink each day. Exclusion criteria: any problem that may have been associated with glucose metabolism or insulin resistance or interfered with the ability of the study participant to follow dietary instructions.	12–16 wk of gestation until delivery (4–7 mo)	Low glycemic index diet (LGI)	The LGI diet was based on previously verified low-GI foods, including pasta and brand-name breads and breakfast cereals with a high-fiber content.	Standard care/high glycemic index (HGI)	Patients were asked to keep their activity or exercise levels the same as usual	Primary: Secondary: GA ↑ BW ↓
Ney et al., 1982 [45]	United States/Not stated	Intervention: 11 Comparator/control: 9	Inclusion criteria: diabetic pregnant women (not gestational diabetes). Exclusion criteria: NA	10–30 wk of gestation until delivery (4–7 mo)	High-carbohydrate, high-fiber diet (HCF)	Daily intake of 60–70 g of dietary fiber: unprocessed wheat bran and/or muffins were prescribed 3 times daily. Type I patients were instructed to eat 3 meals plus snacks at 10:00, at 15:00, and at bedtime, whereas type II patients were counseled to eat 3 meals with a bedtime snack.	Standard diet	Habitual diet	Primary: Secondary: GA ↑ BW ↑
Niinivirta et al., 2011 [46]	Finland/2002–2004	Intervention: 45 Comparator/control: 45	Inclusion criteria: pregnant women from allergic risk families (mother, father or sibling of the unborn child with allergic disease) at <17 wk of gestation were recruited. Exclusion criteria: women presenting severe immunological or other chronic diseases (rheumatoid arthritis, diabetes, inflammatory bowel disease, thyroid diseases, malignancies etc.); who cannot be expected to comply with treatment; who are currently participating or having participated in other clinical trial during the last 2 mo before the beginning of the intervention.	Before 17 wk of gestation until 1 mo postpartum (4–7 mo)	Dietary counseling with intention of increasing the intake of unsaturated fatty acid and reducing that of SFA	The recommended amounts of foods were planned to result in MUFAs contributing 10%–15%, PUFA contributing 5%–10%, and SFA contributing ≤10% of the total daily energy intake. Total intake of fat was aimed for 30%, carbohydrates 55%–60%, and protein 10%–15%. Daily food products with favorable fat compositions (e.g., low-erucic acid rapeseed oil-based spreads and salad dressings) were provided for use at home to support compliance to the recommended diet. Practical dietary advice was adjusted to the women's current dietary habits and food diary analysis.	Standard diet	Habitual diet	Primary: Secondary: GA ↔ BW ↔
Price et al., 2021 [47]	Australia/2014–2019	Intervention: 85 Comparator/control: 79	Inclusion criteria: women with BMI between 30 and 55 who were planning pregnancy within 6 to 12 mo. Exclusion criteria: people who were pregnant, those with known irreversible infertility, diabetes, and/or significant medical illnesses, and those taking medication known to affect weight.	Preconception (6–12 mo) for 12 wk (1–3 mo)	Very low-energy diets (VLED)	Weight loss phase (12 wk): very low-energy diet (∼800 kcal/d), consisting of 2 daily meals of a very low-energy dietary formulation that was provided by the study and a third meal consisting of 2 cups of low starch vegetables, 150 g of lean meat, fish, eggs, or tofu, and 2 teaspoons of oil. Weight maintenance phase (4 wk): dietary counseling to maintain weight. Prepregnancy phase (max 44 wk): counseling to maintain weight.	Standard dietary intervention (SDI)	Energy-reduced diet	Primary: PTB ↔ Secondary:
Reece et al., 1995 [48]	United States/Not stated	Intervention: 24 Comparator/control: 26	Inclusion criteria: women in the first trimester who are patients with insulin-dependent diabetes mellitus (IDDM) or between 24 and 29 wk and have gestational diabetes. Exclusion criteria: subjects in whom the diagnosis of gestational diabetes mellitus was made after 29 wk of gestation were excluded from the study.	First trimester for IDDM and 24–29 wk of gestation for women with GDM until delivery (4–7 mo)	High-fiber diet	Fiber in the fiber-enriched group was administered as 40 g/d in fiber-rich foods (wholegrain bread, cereals, legumes, fruit, and vegetables) and an additional 40 g/d in a high-fiber drink.	Standard American Diabetes Association diet	Soluble fiber mixture derived from oats, beans, peaches, and apples.	Primary: Secondary: GA ↔ BW ↔
Thornton et al., 2009 [49]	United States/1998–2005	Intervention: 124 Comparator/control: 133	Inclusion criteria: women pregnant with a single fetus between 12 and 28 wk of gestation and had a prepregnancy BMI >30 kg/m^2^. Exclusion criteria: patients with pre-existing diabetes, hypertension, or chronic renal disease.	12–28 wk of gestation until delivery (4–7 mo)	Gestational diabetes mellitus (GDM) diet	18–24 kcal/kg balanced nutritional regimen, consisting of 40% carbohydrates, 30% protein, and 30% fat. No patient received a diet of fewer than 2000 calories.	Standard diet	Counseled, at least once, by a registered dietician regarding conventional prenatal nutrition guidelines.	Primary: PTB ↔ Secondary: GA ↔ BW ↔
Van der Maten et al., 1997 [50]	The Netherlands/Not stated	Intervention: 57 Comparator/control: 64	Inclusion criteria: Caucasian, nulliparous women with singleton pregnancies. None of the participating women had a personal history of hypertension. Exclusion criteria: NA	14 wk of gestation until delivery (4–7 mo)	Low sodium (Na) diet	The dietary instructions were based on a dietary Na intake of ∼20 mmol (∼500 mg/d). The women received a brochure listing the products that were allowed or forbidden, a brochure with recipes and a list with the Na content of several products. Practical advice was given regarding shopping locations and commercial products available.	Standard diet	Habitual diet	Primary: Secondary: GA ↔ BW ↔
Vesco et al., 2014 [51]	United States/2009–2011	Intervention: 58 Comparator/control: 60	Inclusion criteria: English-speaking, women with obesity (BMI ≥30 kg/m^2^) aged 18 y or older who were receiving prenatal care at Kaiser Permanente, Northwest. Exclusion criteria: women were excluded if they had diabetes mellitus or other medical conditions requiring specialized nutritional care (e.g., a history of bariatric surgery), or had plans to leave the area during the expected follow-up period (through 1 y postpartum).	15 wk of gestation until delivery (4–7 mo)	DASH diet	Individualized calorie goals and advised them to follow an energy-reduced eating plan based on DASH diet without sodium restriction.	Standard diet	Received a one-time advice session from the study dietician that included general information about eating a healthy diet during pregnancy, without specific focus on the DASH dietary pattern or weight management. Study did not provide any aspect of routine prenatal care for control or intervention participants.	Primary: PTB ↔ Secondary: GA ↔ BW ↔
Walsh et al., 2012 [52]	Ireland/2007–2011	Intervention: 395 Comparator/control: 406	Inclusion criteria: all secundigravid women who had previously delivered a macrosomic infant weighing >4 kg. Exclusion criteria: women with any underlying medical disorders, including a previous history of gestational diabetes, those on any drugs, and those unable to give full informed consent were excluded. Other exclusion criteria were age <18 y, gestation >18 wk, and multiple pregnancy.	Less than 18 wk of gestation until delivery (4–7 mo)	Low glycaemic index diet (LGI)	Attended 1 dietary education session lasting 2 hours in groups of 2 to 6 women with the research dietitian. Advised on general healthy eating guidelines for pregnancy, following the food pyramid. The remainder of the education session focused on the glycaemic index its definition, concept, and rationale for use in pregnancy. Women were encouraged to choose as many low glycaemic index foods as possible and to exchange high glycaemic index carbohydrates for low glycaemic index alternatives. Women received written resources about low glycemic index foods after the education session (web appendix). The recommended low glycaemic index diet was eucaloric, and women were not advised to reduce their total caloric intake.	Standard diet	Habitual diet	Primary: PTB ↔ Secondary: BW ↔
Xu et al., 2023 [53]	China/2020–2022	Intervention: 128 Comparator/control: 123	Inclusion criteria: include pregnant women aged 20 y or older and before 20 wk of gestation with 1 or more risk factors for GDM, such as age ≥35 y, prepregnancy BMI ≥ 25 kg/m^2^, family history of first-degree diabetes, history of polycystic ovary syndrome (PCOS), GDM, and history of adverse births such as macrosomia, miscarriage, and preterm birth. Exclusion criteria: type 2 diabetes mellitus, hypertension, hypothyroidism, multiple pregnancies, severely limited food choices, failure to guarantee the number of prenatal check-ups, presence of severe mental disorders, communication disorders, history of antipsychotic use, and history of adverse lifestyle such as smoking, alcohol consumption, and toxic exposure in the past 3 mo.	<20 wk of gestation until delivery (4–7 mo)	Prenatal nutrition to control gestational weight gain	Nutritional recommendations were individualized and could be given through the food exchange method, taking into account the preferences and dietary needs of the pregnant women without exceeding the total energy intake. Encouraged to eat a balanced diet with vegetables, fruits, high-fiber whole grain products, low-fat dairy products, increased legumes, nuts and other plant proteins, and other plant proteins whereas avoiding foods rich in sugar and SFAs.	Standard diet	Habitual diet	Primary: PTB ↔ Secondary:
Yao et al., 2015 [54]	China/2014–2014	Intervention: 18 Comparator/control: 19	Inclusion criteria: women aged 18–40 y, gravid for the first time and diagnostic of gestational diabetes by means of a 100-g oral glucose tolerance test during pregnancy for 24–28 wk, were enlisted in this study. Exclusion criteria: NA	24–28 wk of gestation for 4 wk (1–3 mo)	DASH diet	45%–55% carbohydrates, 15%–20% protein and 25%–30% total fat. Abundant in fruits, vegetables, whole grains, and dairy products with low fat, and low in saturated fats, cholesterol, refined grains, and sweets. The daily intake of sodium was 2400 mg. The DASH diet founded on 2000 kcal. 400 mg/d folic acid from the outset of gestation and 50 mg/d ferrous sulfate as well as multivitamin-mineral supplements from 20 wk of gestation.	Standard diet	45%–55% carbohydrates, 15%–20% protein and 25%–30% total fat. 400 mg/d folic acid from the outset of gestation and 50 mg/d ferrous sulfate as well as multivitamin-mineral supplements from 20 wk of gestation.	Primary: Secondary: GA ↔ BW ↓

Ongoing RCTs									

ACTRN12624000936527 [55]		Australia/2024-ongoing	Inclusion criteria: Females scheduled for in vitro fertilization (with or without intracytoplasmic sperm injection), with oocyte pickup following the intervention. Participation of the male partner in the dietary intervention will be strongly encouraged, but not mandatory. Female aged 35–43 y. No limit for the age of the male partner. Use of partner sperm. Fresh embryo transfer, or frozen to be transferred within 3 mo Exclusion criteria: polycystic ovary syndrome. Use of donor oocytes. Medical contraindication to in vitro fertilization. Weight change of >3 kg in the 6 mo before recruitment. Use of frozen sperm. More than 3 previous unsuccessful in vitro fertilization attempts. Preimplantation genetic testing. Mediterranean Diet Adherence Score (MEDAS) >10 at baseline. Not willing to be randomly assigned to the diet.	8 wk before IVF treatment (<8 mo)	Mediterranean diet	Eating mainly plant-based foods (e.g., fruits, vegetables, herbs, spices, and whole grains) as well as moderate amounts of fish, dairy, and poultry, with the use of olive oil as the main source of dietary fat, and limiting the intake of red meat and added sugar.	Standard diet (dietary intervention in accordance with the Australian Dietary Guidelines)	Eating a wide variety of healthy foods (e.g., fruits, vegetables, dairy, lean meats, whole grains) and limiting the intake of foods high in saturated fat, salt and added sugar.	Primary: Secondary: GA, BW
Meloncelli et al., 2023 [56]		Australia/2022-ongoing	Inclusion criteria: pregnant women, 18 y or older, with a singleton pregnancy are eligible if they enroll in the study before 18 wk of gestation, and have 1 or more of the following risk factors for developing GDM: history of GDM; a first-degree relative with type 2 diabetes mellitus or a mother/sister with a history of GDM; prepregnancy BMI >25 kg/m^2^; or a biological child who weighed >4000 g at birth. Exclusion criteria: women will be excluded if they have a confirmed diagnosis of any form of diabetes (including GDM) at the time of enrolment. Due to the complex or confounding nature of certain conditions, women with reported cystic fibrosis, inflammatory bowel disease, bariatric surgery or bowel disorder requiring a restrictive diet, a diagnosed eating disorder, or other complex medical comorbidities (e.g., kidney or heart disease) will also be excluded. Assisted conception (e.g., in vitro fertilization) is not an exclusion criterion. Women who are unable to understand the intervention (e.g., have insufficient understanding of spoken English).	11–18 wk of gestation until delivery (4–7 mo)	Healthy gut diet	Fiber-rich, prebiotic whole foods found exclusively in vegetables, fruit, whole grains, legumes/lentils, nuts, and seeds. Include a variety of plant foods (minimally processed) at each meal; Reduce the intake of ultraprocessed foods and foods high in saturated fat; eat naturally fermented foods.	Standard diet	Standard diet	Primary: PTB Secondary: GA
Coppola et al., 2022 [57]		Italy/2022-ongoing	Inclusion criteria: all healthy women (aged 20–35 y) attending their first pregnancy visit at 8–13 gestational wk consecutively observed at the 4 centers. We will consider only pregnant women with a pregnancy of at risk for atopy infant. Exclusion criteria: all women aged <20 and >35 y, or with concomitant presence of infections, malignancies, major gastrointestinal malformations, immunodeficiencies, autoimmune diseases, chronic inflammatory bowel diseases, pre-existing diabetes, chronic renal disease, celiac disease, history of abdominal surgery with gastrointestinal resection, neurologic and neuropsychiatric disorders, genetic and metabolic disorders, vegan diet, twin pregnancy.	8–13 wk of gestation until delivery (>8 mo)	Mediterranean diet	Use of extra virgin olive oil as main cooking fat (≥4 tablespoons/d), high intake of vegetables (2 servings/d), fruits (3 servings/d) (avoiding juices) and wholegrain cereals (3 servings/d), 3 servings of skimmed dairy products/d, 2–3 servings of legumes/wk, 3 servings of fish/wk, 3 servings of nuts and seeds/wk, drink ≥2 L of water daily, a low consumption of red meat and processed meat, avoidance of refined grains, processed baked goods, presliced bread, soft drinks and fresh juices, fast foods, and precooked meals.	Standard diet	Standard diet	Primary: PTB Secondary:
Dapre et al., 2024 [58]		The UK/2022-ongoing	Inclusion criteria: pregnant women ≥18 y, BMI ≥27.5 kg/m^2^ or a BMI ≥25 kg/m^2^ in high-risk minority ethnic group (i.e., South Asian, Black African, African Caribbean) and <50 kg/m^2^ at booking appointment (8–12 wk of gestation). Newly diagnosed GDM according to local diagnostic criteria (fasting glucose ≥5.3 mmol/L and/or 2-hour postprandial glucose ≥8.5 mmol/L in a 75 g OGTT) scheduled to receive first-line diet and physical activity (best NHS care), 24–30 wk pregnant at screening appointment. Exclusion criteria: pregestational type 1 or type 2 diabetes. Fasting glucose of ≥7 or 2-hour postprandial of ≥11 on OGTT (immediate intervention with medication would be required in this group of women). Current multiple pregnancy Maturity Onset Diabetes of the Young. Significant comorbid disease that in PI's opinion would preclude participation in the study, for example, chronic kidney disease, significant cardiac disease, history of disordered eating or severe psychological problems. Current participation in a GDM medication treatment trial. People who are not capable of providing informed consent or adhering to the monitoring and safety protocols. People who have previously had bariatric surgery for weight loss including gastric bypass and sleeve gastrectomy, and/or those prescribed weight loss medications (e.g., orlistat). Medications at the time of the OGTT that may interfere with results (e.g., high dose oral steroids, immunosuppressants) Previous history of intrauterine growth restriction. Women who have lost >5% of their weight from booking appointment to screening appointment.	24–30 wk of gestation until delivery (4–7 mo)	Intermittent low-energy diet	Several days of a food-based or meal replacement (e.g., drinks/bars) low-energy diet (650–1000 kcal), with a standard healthy (non-restrictive) diet recommended on the remaining days of the week.	Control	Best National Health Service care in GDM	Primary: Secondary: GA
Herman et al., 2024 [59]		United States/2021-ongoing	Inclusion criteria: prepregnancy BMI 18.5–45.0 kg/m^2^ Exclusion criteria: pregestational diabetes, hypothyroidism, hyperemesis gravidarum, eating disorder, asthma, heart attack, current smoker, drug abuse, psychological disorder, recent antibiotic use, and pregnancies of multiples.	18 wk (4–7 mo)	High-fiber diet	Snacks totaling 10–12 g/d of fiber (210–380 kcal/d)	Standard diet	Standard diet	Primary: Secondary: GA
NCT05868954 [60]		United States/2023-ongoing	Inclusion criteria: viable singleton pregnancy in the first trimester (6 0/7–16 6/7 wk); includes twins reduced to singleton spontaneously or vanishing twin syndrome. BMI ≥ 25.0 kg/m^2^; calculated by dividing maternal weight in kilograms by height in meters squared using a calibrated scale and standard metric measure. Confirmed intrauterine pregnancy by ultrasound examination (6–16 wk) Age 18 y or older. Primary language of English or Spanish. Exclusion criteria: BMI <25.0 kg/m^2^. Known prepregnancy diabetes, hemoglobin glycosylated (A1C) >5.7% at first prenatal visit. Prepregnancy hypertensive disease. Nonviable pregnancy. Known allergies to an essential component(s) of MedDiet. Inability to read or write in primary language. Mental incapacity to make medical decisions.	6 0/7–16 6/7 wk of gestation until delivery (4–7 mo)	Mediterranean diet	Well-known healthy diet that consists of a large amount of plant-based foods such as fruits, vegetables, beans, and nuts with extra virgin olive oil (EVOO) as the principal source of fat. Dairy, fish, and poultry are consumed in moderation and red meat only eaten occasionally.	Control	American College of Obstetricians and Gynecologists (ACOG)-based Dietary Program	Primary: PTB Secondary:
NCT06614413 [61]		Spain/2024-ongoing	Inclusion criteria: fluency in the Spanish language, singleton pregnancy. Exclusion criteria: children conceived with assisted reproduction techniques. Fetal anomalies and congenital infections. Neonatal anomalies diagnosed after birth. Intellectual development disorder, due to clinical judgment, inability to attend visits. Participation in another controlled trial. Vegetarian diet.	4–14 wk of gestation until delivery (4–7 mo)	Mediterranean diet	Not indicated	Control	Not indicated	Primary: Secondary: BW
Palmer et al., 2022 [62]			Inclusion criteria: <23 wk of gestation. Women able to give informed consent, a singleton pregnancy and women who are planning to breast feed for ≥4 mo. The fetus is to have ≥2 biological family members (mother, father or siblings) with medically diagnosed allergic disease (asthma, eczema, hay fever, or IgE-mediated food allergy). Exclusion criteria: women with egg or peanut allergies, as they would be unable to safely follow the intervention without allergic reactions.	<23 wk of gestation until 4 mo of postnatal infant age (>8 mo)	Diet rich in eggs and peanuts	Regular maternal consumption of ≥6 eggs and 60 peanuts per week	Control	Standard (low) egg and peanut diet group	Primary: Secondary: BW, GA
Waselewski et al., 2022 [63]		United States/2021-ongoing	Inclusion criteria: gestational age ≤20 wk. Text message capability. Healthy singleton pregnancy. Nulliparous. Consume sugar-sweetened beverages. Living within delivery zone of a grocery delivery service. Exclusion criteria: non-English speaking. Participants who live at the same address. Physical, mental, or cognitive handicaps that prevent participation. High-risk pregnancy requiring specialized care (including pre-existing diabetes).	Less than 20 wk of gestation until delivery (4–7 mo)	Intervention 1: healthy diet grocery delivery Intervention 2: healthy diet grocery delivery + unsweetened beverage delivery	Intervention 1: each food delivery will contain ∼$35 worth of fresh fruits, vegetables, dairy products, and whole grain foods. These foods are not meant to supplant regular meals, rather make healthy eating more convenient. Intervention 2: each food delivery will contain ∼$35 worth of fresh fruits, vegetables, dairy products, and whole grain foods. These foods are not meant to supplant regular meals, rather make healthy eating more convenient + unsweetened beverages to replace normal sugar-sweetened beverage intake	Control	Standard diet	Primary: Secondary: BW

Abbreviations: ADA, American Diabetes Association; BEP, balanced energy protein; BW, birthweight; CHO, carbohydrate; DASH, dietary approaches to stop hypertension; FFS, fortified food supplement; GA, gestational age; GD/GDM, gestational diabetes mellitus; GH, gestational hypertension; GI, glycaemic index; IVF, in-vitro fertilization; LBW, low birthweight; MMN, multiple micronutrient; OGTT, oral glucose tolerance test; PTB, preterm birth; RCT, randomized controlled trial; Ref, reference; TEI, total energy intake.

### Grading the certainty of evidence

Two reviewers (CS and AB) independently assessed the certainty of evidence for the 2 primary outcomes (preterm birth and low birthweight) using the Grading of Recommendations Assessment, Development and Evaluation (GRADE) approach [64]. We evaluated the certainty of evidence as high, moderate, low, or very low across the 5 domains [risk of bias (based on the quality of evidence), inconsistency (based on the heterogeneity *I*^2^), indirectness (based on results measuring the exposure and outcome of interest), imprecision (based on sample sizes), and publication bias (based on visual inspection of funnel plots)] and presented our findings in a “Summary of findings” table using the GradePro Guideline Development Tool [65].

## Results

### Literature search and study selection process

**FIGURE 1:**
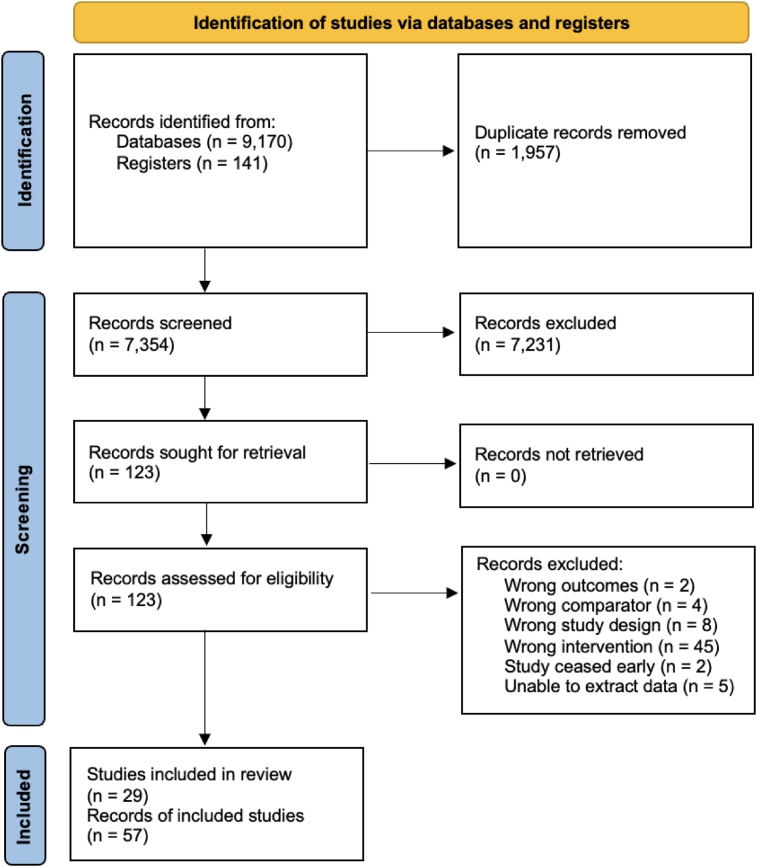
Flow of literature.

Overall, 9311 records were identified from the initial database and reference list searches; 123 full-text records were assessed for eligibility of which 45 were excluded (Figure 1). Finally, 29 studies [with 49 records of included studies (publications and trial registrations)] met the inclusion criteria and were included in the final review and quantitative analysis [[26], [27], [28], [29], [30], [31], [32], [33], [34], [35], [36], [37], [38], [39], [40], [41], [42], [43], [44], [45], [46], [47], [48], [49], [50], [51], [52], [53], [54]]. Nine (2 merged reports on the same RCT) are ongoing RCTs and included in the final review. The list of the 45 records excluded via full-text assessment and the reasons for exclusions is provided in Supplemental Table 2.

### Characteristics of primary trials included in the meta-analysis

Table 1 contains the general characteristics of all 29 included RCTs and the 9 ongoing RCTs, and a descriptive explanation of all dietary pattern interventions included in the meta-analysis. To summarize, the analysis included a total of 7367 participants who were pregnant or planning pregnancy in an outpatient setting from 15 different countries. The geographic distribution of the 29 RCTs included 20 high-income [28,30,[34], [35], [36], [37], [38],[40], [41], [42], [43], [44], [45], [46], [47], [48], [49], [50], [51], [52]], 7 upper-middle-income [[26], [29],^31^,^32^,^39^,^53^,^54^], 2 lower-middle-income [27,33], and no low-income countries based on the 2024 World Bank Classification [25].

All 29 RCTs compared dietary interventions before and/or during pregnancy with placebo/control/standard care on birth outcomes, including preterm birth [27,28,[30], [31], [32],^34^,^36^,^39^,^40^,^41^,[47], [49],[51], [52], [53]], low birthweight [[26], [31], [32], [33],^36^,^39^,^41^], different severity of preterm birth [28,31,51,52], mean gestational age [[26], [27],^29^,^30^,^32^,^36^,^38^,^39^,[40], [41], [42], [43], [44], [45], [46],[48], [49], [50], [51], [54]], and mean birthweight [[27], [28], [29], [30], [31], [32], [33],[35], [36], [37], [38], [39], [40], [41], [42],[44], [45], [46],[48], [49], [50], [51], [52],^54^]. Of the RCTs, 1 began before conception [47], 4 RCTs began the dietary intervention in the first trimester (≤12 wk of gestation) [[26], [30],^33^,^35^,], 10 RCTs began the dietary intervention in the second trimester of pregnancy (13–27 wk of gestation) [[28], [31],^34^,^39^,^41^,^46^,[50], [51], [52], [53]], 4 RCTs began the dietary intervention in the third trimester of pregnancy (28–40 wk of gestation) [37,38,40,43], and 10 RCTs were not within a defined timeframe (no specific trimester or across several trimesters) [27,29,32,36,42,44,45,48,49,54]. The median intervention duration was 4 to 7 mo (from 1 to > 9 mo), with 10 RCTs < 4 mo [27,29,32,38,39,40,42,43,[47], [54]]. The median sample size was 98 participants in the intervention group and 93 in the comparator group (from 9 to 579 participants). Participants with varying weight status and gestational metabolic conditions participated in the RCTs. Four RCTs focused solely on participants with overweight/obesity (BMI ≥25 kg/m^2^) [[36], [47], [49],^51^], and none addressed participants who were underweight (BMI <18.5 kg/m^2^). The remaining RCTs included participants of mixed bodyweight. Additionally, 9 RCTs exclusively enrolled participants with gestational diabetes mellitus (GDM) [27,29,32,38,40,42,43,45,54], whereas 13 RCTs included participants with and without GDM [28,30,[35], [36], [37],^39^,[41], [47],^48^,^49^,[51], [52], [53]]. Finally, 5 RCTs included some participants with gestational hypertension (GH) [28,30,[39], [47],^49^].

### Risk of bias or quality of included studies

**FIGURE 2:**
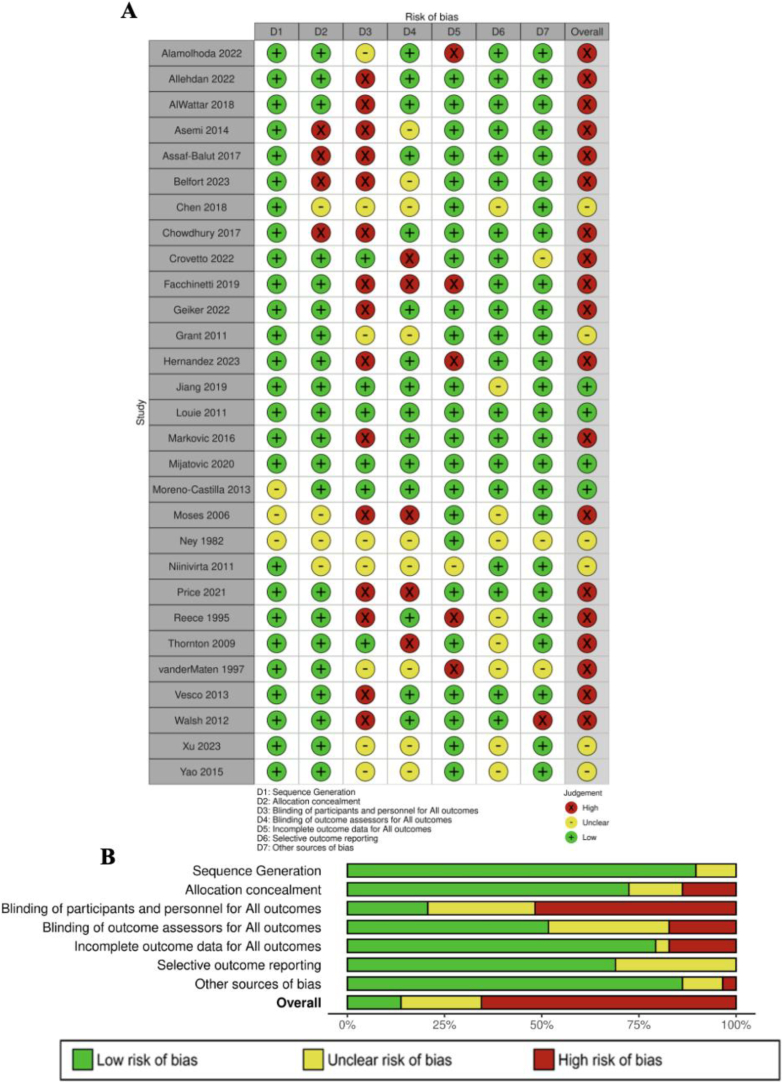
Risk of bias assessment. (A) Risk of bias summary “traffic light” plot of each result's domain-level judgments. (B) Risk of bias weighted bar plot using Cochrane Risk of Bias tool I: review authors’ judgments about each risk of bias item presented as percentages across all included studies.

Figure 2 demonstrates the overall low methodological quality based on the quality assessment of the 29 RCTs. Of these, 5 (17%) had an unclear risk of selection bias due to insufficient information on sequence generation and/or allocation concealment and 4 (14%) had a high risk of selection bias due to no allocation concealment. Eight RCTs (28%) had an unclear risk of performance bias due to insufficient information regarding the blinding of the participants and personnel, and 15 (52%) had a high risk of performance bias because of the lack of blinding of participants and/or personnel. Nine (31%) RCTs had an unclear risk of detection bias due to unclear information on the blinding of outcome assessors, and 5 (17%) had a high risk of detection bias. There were 1 (3%) RCTs with an unclear risk of attrition bias, and 5 (17%) had a high risk of attrition bias due to a high loss to follow-up or no description given for the dropouts/loss to follow-up. There were 9 (31%) RCTs with an unclear risk of reporting bias, and the rest were low risk. Finally, 3 (10%) had unclear other sources of bias, whereas 1 (3%) had a high risk of other sources of bias due to imbalances in prognostic characteristics and potential disruption of intervention supplies to the participants.

### Primary outcome: preterm birth

**FIGURE 3:**
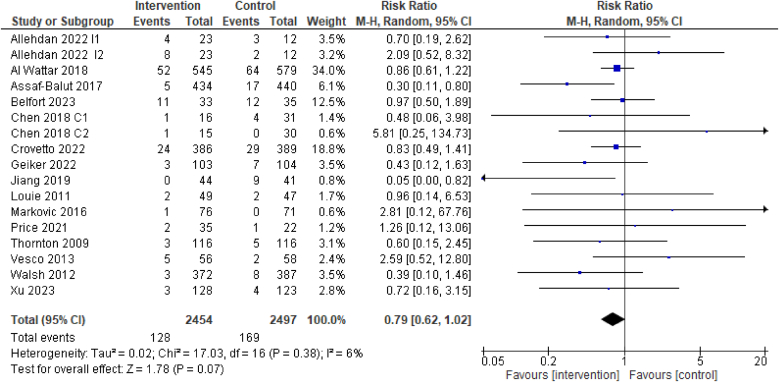
Effect of dietary intervention on preterm birth. Results from randomized or quasi-randomized controlled trials. The diamond characterizes the overall effect estimate. Data are presented as a risk ratio with 95% CIs, using the random-effects model. CI, confidence interval; df, degrees of freedom; M-H, Mantel-Haenszel.

For the primary outcome of preterm birth, evidence from 15 RCTs suggests that dietary interventions of improved diet quality before and/or during pregnancy may reduce the incidence of preterm birth, though this was not statistically significant [15 RCTs, 4949 participants, RR 0.79 (0.62, 1.02), *I*^2^ = 6%, low certainty of evidence, Figure 3]. Two RCTs with interventions that entailed following specific dietary patterns found a lower incidence of preterm birth compared with the control/comparator: Mediterranean diet [RR 0.30 (0.11, 0.80)] and DASH diet [RR 0.05 (0.00, 0.82)] [30,39].

### Primary outcome: low birthweight

**FIGURE 4:**
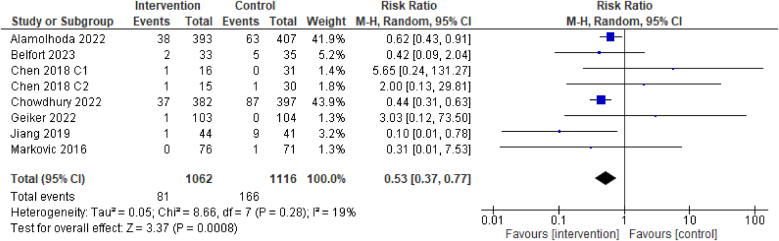
Effect of dietary intervention on low birthweight. Results from randomized or quasi-randomized controlled trials. The diamond characterizes the overall effect estimate. Data are presented as a risk ratio with 95% CIs, using the random-effects model. CI, confidence interval; df, degrees of freedom; M-H, Mantel-Haenszel.

For the primary outcome of low birthweight, evidence from the 7 RCTs suggests dietary interventions of improved diet quality before and/or during pregnancy may result in a reduction in the incidence of a low birthweight infant [7 RCTs, 2178 participants, RR 0.53 (0.37, 0.77), *I*^2^ = 19%, low certainty of evidence, Figure 4]. Three RCTs found a lower incidence of low birthweight in the trials with interventions of the following dietary patterns compared with the control/comparator: low trans fatty acid diet [RR 0.62 (0.43, 0.91)]; balanced plate diet (intake of diversified food-yielding ≥2500 kcal energy per day) [RR 0.44 (0.31, 0.63)], and DASH diet [RR 0.1 (0.01, 0.78)] [[26], [33],^39^].

### Secondary outcomes

#### Severity of preterm birth

**FIGURE 5:**
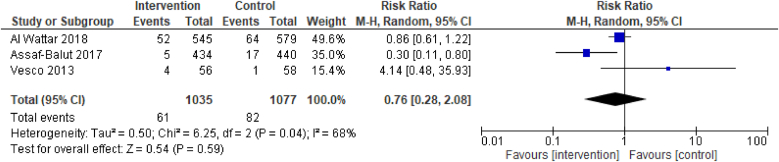
Effect of dietary interventions on late preterm birth. Results from randomized or quasi-randomized controlled trials. The diamond characterizes the overall effect estimate. Data are presented as a risk ratio with 95% CIs, using the random-effects model. CI, confidence interval; df, degrees of freedom; M-H, Mantel-Haenszel.

Evidence from 3 RCTs demonstrated that dietary interventions of improved diet quality before and/or during pregnancy have no effect on the outcome of late preterm birth, between 34^+0^ and <37 wk of pregnancy [3 RCTs, 2112 participants, RR 0.76 (0.28, 2.08), *I*^2^ = 68%, Figure 5].

**FIGURE 6:**
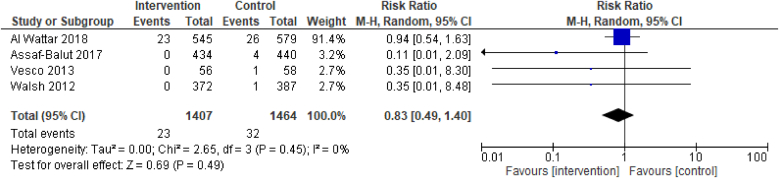
Effect of dietary interventions on moderately preterm birth. Results from randomized or quasi-randomized controlled trials. The diamond characterizes the overall effect estimate. Data are presented as a risk ratio with 95% CIs, using the random-effects model. CI, confidence interval; df, degrees of freedom; M-H, Mantel-Haenszel.

Evidence from 4 RCTs demonstrated that dietary interventions of improved diet quality before and/or during pregnancy have no effect on the outcome of moderately preterm birth, born between 32^+0^ and <34 wk of pregnancy [4 RCTs, 2871 participants, RR 0.83 (0.49, 1.40), *I*^2^ = 0%, Figure 6].

Only 1 RCT reported the outcome of very preterm birth, between 28^+0^ and <32 wk of pregnancy, but did not calculate any associations with the dietary intervention [52].

#### Gestational age (mean, in weeks)

**FIGURE 7:**
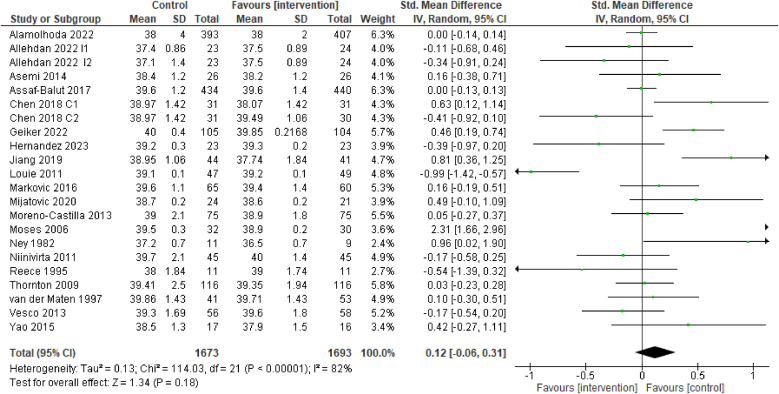
Effect of dietary interventions on mean gestational age in weeks. Results from randomized or quasi-randomized controlled trials. The diamond characterizes the overall effect estimate. Data are presented as a std mean difference with 95% CIs, using the random-effects model. IV, inverse variance; Std., standard.

The evidence suggests that dietary interventions of improved diet quality before and/or during pregnancy have no effect on mean gestational age at birth [20 RCTs, 3366 participants, SMD 0.12 (–0.06, 0.31) wk, *I*^2^ = 82%, Figure 7]. The SMD was used due to the different methods employed by the RCTs to measure gestational age (recall of date of last menstrual period compared with ultrasound).

#### Birthweight (mean, in grams)

**FIGURE 8:**
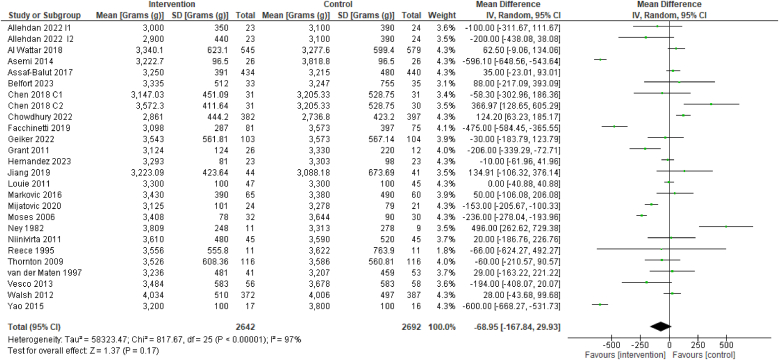
Effect of dietary interventions on mean birthweight in grams (g). Results from randomized or quasi-randomized controlled trials. The diamond characterizes the overall effect estimate. Data are presented as a mean difference with 95% CIs, using the random-effects model. IV, inverse variance; Std., standard.

The evidence suggests that dietary interventions of improved diet quality before and/or during pregnancy have no effect on the mean birthweight [24 RCTs, 5334 participants, SMD –68.95 (–167.84, 29.93) g, *I*^2^ = 97%, Figure 8].

### Subgroup and stratified analyses

We further analyzed the data to determine whether the effect we found for the primary outcomes (preterm birth and low birthweight) differed for specific subgroups and to explore the low heterogeneity demonstrated by the primary outcomes (preterm birth *I*^2^ = 6%; low birthweight *I*^2^ = 19%) (Table 2). Due to insufficient data, we were unable to undertake other preplanned subgroup analyses of the sex of the infant (male compared with female) and maternal nutritional status (study-defined). We were also unable to conduct sensitivity analyses for the primary outcomes by excluding RCTs at high risk of bias, as we only judged 1 study to be high-quality for low birth weight and 2 for preterm birth.

**TABLE 2:** Summary of subgroup analysis.

Outcome	Subgroups	No. of participants (studies)	Relative risk (95% CI)	*P* for overall effect	*I*^2^ (%)	*P* for subgroup interaction
Duration of dietary intervention						
Preterm birth	<1 mo	0	—	—	—	0.79
	1–3 mo	400 (5)	0.89 (0.38, 2.10)	0.79	26	
	4–7 mo	4551 (10)	0.79 (0.62, 1.01)	0.06	2	
	8–9 mo	0	—	—	—	
	>9 mo	0	—	—	—	
Low birthweight	<1 mo	0	—	—	—	0.7
	1–3 mo	177 (2)	0.86 (0.07, 10.89)	0.9	65	
	4–7 mo	2001 (5)	0.52 (0.40, 0.67)	<0.00001	0	
	8–9 mo	0	—	—	—	
	>9 mo	0	—	—	—	
Risk						
Preterm birth	Low/no risk	874 (1)	0.30 (0.11, 0.80)	0.02	—	0.04
	High risk	4077 (14)	0.85 (0.68, 1.07)	0.17	0	
Low birthweight	Low/no risk	1579 (2)	0.52 (0.37, 0.73)	0.0002	41	0.76
	High risk	599 (5)	0.64 (0.19, 2.10)	0.46	29	
Timing of dietary interventions						
Preterm birth	Preconception	57 (1)	1.26 (0.12, 13.06)	0.85	—	0.39
	First trimester	874 (1)	0.30 (0.11, 0.80)	0.02	—	
	Second trimester	3323 (8)	0.85 (0.62, 1.15)	0.29	14	
	Third trimester	96 (1)	0.96 (0.14, 6.53)	0.97	—	
	Across trimesters or no specific trimester	601 (4)	0.80 (0.43, 1.52)	0.5	0	
Low birthweight	Preconception	0	—	—	—	0.06
	First trimester	1579 (2)	0.52 (0.37, 0.73)	0.0002	41	
	Second trimester	752 (4)	0.26 (0.08, 0.82)	0.02	0	
	Third trimester	0	—	—	—	
	Across trimesters or no specific trimester	299 (2)	3.08 (0.55, 17.30)	0.2	0	
Type of dietary patterns						
Preterm birth	Mediterranean/DASH diet	3075 (7)	0.82 (0.53, 1.28)	0.39	51	0.75
	High-fiber diet	0	—	—	—	
	Low-carb/sugar diet	1568 (7)	0.62 (0.35, 1.12)	0.11	0	
	Other/undefined (focused) diet	308 (2)	0.84 (0.24, 2.94)	0.79	0	
Low birthweight	Mediterranean/DASH diet	153 (2)	0.24 (0.06, 0.97)	0.05	19	0.15
	High-fiber diet	0	—	—	—	
	Low-carb/sugar diet	446 (3)	1.83 (0.40, 8.36)	0.43	0	
	Other/undefined (focused) diet	1579 (2)	0.52 (0.37, 0.73)	0.0002	41	
Macronutrient-specific interventions						
Preterm birth	Low-carb	1393 (7)	0.66 (0.35, 1.23)	0.19	0	0.77
	High-carb	0	—	—	—	
	High-fat-quality/unsaturated fats	2773 (3)	0.72 (0.45, 1.13)	0.16	51	
	Recommended macronutrient intake	534 (5)	0.97 (0.41, 2.31)	0.94	50	
Low birthweight	Low-carb	446 (3)	1.83 (0.40, 8.36)	0.43	0	0.08
	High-carb	0	—	—	—	
	High-fat-quality/unsaturated fats	800 (1)	0.62 (0.43, 0.91)	0.01	—	
	Recommended macronutrient intake	932 (3)	0.42 (0.30, 0.60)	<0.00001	0	
Region						
Preterm birth	High-income	4385 (10)	0.78 (0.60, 1.00)	0.05	0	0.74
	Upper-middle-income	496 (4)	0.70 (0.26, 1.88)	0.48	41	
	Lower-middle-income	70 (1)	1.18 (0.40, 3.49)	0.76	22	
	Low-income	0	—	—	—	
Low birthweight	High-income	354 (2)	0.97 (0.10, 9.23)	0.98	0	0.69
	Upper-middle-income	1045 (4)	0.58 (0.25, 1.33)	0.2	32	
	Lower-middle-income	779 (1)	0.44 (0.31, 0.63)	<0.00001	—	
	Low-income	0	—	—	—	

Abbreviations: Carb, carbohydrate; CI, confidence interval; DASH, dietary approaches to stop hypertension.

#### Duration of dietary intervention

There was no significant interaction between the duration of dietary intervention and the intervention effect on preterm birth (*P* = 0.79 for interaction, Supplemental Figure 1) or low birthweight (*P* = 0.7 for interaction, Supplemental Figure 2). However, the evidence suggests that dietary interventions lasting 4–7 mo showed a protective effect against low birthweight [5 RCTs, 599 participants, RR 0.52 (0.40, 0.67)], whereas no effect was observed for durations of 1–3 mo (Supplemental Figure 2). Data were predominantly available for intervention durations of 1–7 mo, with no data reported for durations <1 mo, between 8 and 9 mo, or exceeding 9 mo for either outcome (Supplemental Table 3).

#### Baseline risks

There was a significant interaction between the baseline risk of preterm birth and dietary interventions (*P* = 0.04 for interaction, Supplemental Figure 3), but not for the baseline risk of low birthweight (*P* = 0.76 for interaction, Supplemental Figure 4). Women in the intervention group had a significantly reduced incidence of preterm birth if they were at a prior low risk [1 RCT, 874 participants, RR 0.30 (0.11, 0.80)] of having a preterm birth at baseline compared with the women in the control/comparator group based on evidence from 1 RCT. Women in the intervention groups had a reduced incidence of low birthweight compared with women in the control/comparator group if they were at a prior low risk [2 RCTs, 1579 participants, RR 0.52 (0.37, 0.73)] of having a low birthweight infant compared with the women in the control/comparator group (Supplemental Table 4).

#### Timing of dietary intervention

There was no significant interaction between the timing of dietary intervention and the intervention effect on preterm birth (*P* = 0.39, Supplemental Figure 5) or low birthweight (*P* = 0.06 for interaction, Supplemental Figure 6). For preterm birth, evidence from 1 RCT suggested that women who initiated the intervention in the first trimester had a lower incidence compared with the control group [1 RCT, 874 participants, RR 0.30 (0.11, 0.80)], whereas no effect was observed for interventions starting preconception, in the second or third trimesters, or across trimesters/no specific trimester. For low birthweight, a reduced risk was seen when interventions began in the first [2 RCTs, 1579 participants, RR 0.52 (0.37, 0.73)] or second trimester [4 RCTs, 752 participants, RR 0.26 (0.08, 0.82)], whereas no effect was observed for interventions spanning across trimesters or without a clearly defined timing. Data were primarily available for interventions initiated during the second trimester or spanning trimesters for both outcomes, whereas no data were reported for interventions beginning preconception or in the third trimester with respect to low birthweight.

#### Type of dietary patterns

There was no significant interaction between the type of dietary patterns implemented and the intervention effect on preterm birth (*P* = 0.75 for interaction, Supplemental Figure 7) or low birthweight (*P* = 0.15 for interaction, Supplemental Figure 8). For the outcome of low birthweight, women who adhered to the dietary patterns prioritizing Mediterranean/DASH diets [2 RCTs, 153 participants, RR 0.24 (0.06, 0.97)] or other focused diets [low trans-fatty acid diet and balanced plate nutrition; 2 RCTs, 1579 participants, RR 0.52 (0.37, 0.73)] had a lower incidence of low birthweight compared with the control/comparator group, whereas there was no effect for interventions of low-carb/sugar diet or other/undefined (focused) diets. Data for the high-fiber diet were unavailable for both outcomes.

#### Type of macronutrient-specific interventions

**FIGURE 9:**
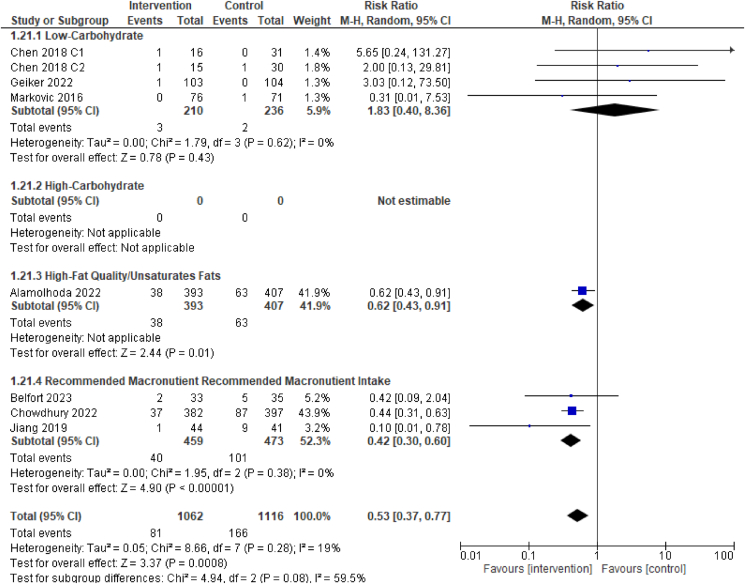
Effect of macronutrient-specific dietary interventions on low birthweight. Results from randomized or quasi-randomized controlled trials. The diamond characterizes the overall effect estimate. Data are presented as a risk ratio with 95% CIs, using the random-effects model. CI, confidence interval; df, degrees of freedom; M-H, Mantel-Haenszel.

There was no significant interaction between the macronutrient-specific interventions and the intervention effect on preterm birth (*P* = 0.77 for interaction, Supplemental Figure 9) or low birthweight (*P* = 0.08 for interaction, Figure 9). Women who received dietary interventions providing high-fat quality/unsaturated fat diets [low trans-fatty acid diet; 1 RCT, 800 participants, RR 0.62 (0.43, 0.91)] based on evidence from 1 RCT, or the recommended macronutrient intake diets [DASH and balanced plate nutrition diets; 3 RCTs, 932 participants, RR 0.42 (0.3, 0.6)] had a lower incidence of low birthweight compared with the women in the control/comparator group, whereas there was no effect for dietary interventions promoting low-carbohydrate diets. Data for the high-carbohydrate diet were unavailable for both outcomes.

#### Socioeconomic status of study region

There was no significant interaction between the socioeconomic status of the study region where the dietary interventions took place and the intervention effect on preterm birth (*P* = 0.74 for interaction, Supplemental Figure 10) or low birthweight (*P* = 0.69 for interaction, Supplemental Figure 11). In high-income countries, dietary interventions were associated with a lower incidence of preterm birth compared with control groups [10 RCTs, 4385 participants, RR 0.78 (0.60, 1.00)], whereas no effect was observed in upper-middle- or lower-middle-income settings. For low birthweight, an effect was observed in 1 RCT conducted in a lower-middle-income country [1 RCT, 779 participants, RR 0.44 (0.31, 0.63)], whereas no effect was found in high- or upper-middle-income regions. Data from low-income countries were unavailable for both outcomes.

#### Stratified analysis

An exploratory stratified meta-analysis was conducted by grouping 4 geographical regions of the included RCTs with dietary interventions providing the recommended macronutrient intake or high-fat quality/unsaturated fats into 2 categories (high-/upper-middle-income and low-/lower-middle-income). There was no significant interaction between region subgroups and the intervention effect on preterm birth (*P* = 0.25, Supplemental Figure 12) or low birthweight (*P* = 0.99, Supplemental Figure 13). Dietary interventions providing recommended macronutrient intake or high-fat-quality/unsaturated fat diets were associated with a reduced overall risk of low birthweight [RR 0.44 (95% CI: 0.32, 0.61)]. Both high-/upper-middle-income [RR 0.44 (0.19, 1.04)] and low-/lower-middle-income regions [RR 0.44 (0.31, 0.63)] showed directionally consistent effects. However, the evidence was limited by the small number of included trials (3 in higher-income settings and 1 in a lower-middle-income setting) and small sample sizes.

### Publication bias

Visual inspection of the funnel plot for the outcomes with >10 RCTs indicates no asymmetry for outcomes of preterm birth, mean gestational age, and mean birthweight (Supplemental Figures 14–16).

### Certainty of evidence (GRADE assessment)

The GRADE assessment (“Summary of Findings” table) is presented in Table 3. The effect of dietary interventions before and/or during pregnancy was graded as “low” certainty of evidence for the outcomes of preterm birth and low birthweight.

**TABLE 3:** GRADE assessment.

Outcomes	No. of participants (studies)	Certainty of the evidence (GRADE)	Relative effect (95% CI)	Anticipated absolute effects	
				Risk with placebo/standard care	Risk difference with specified diet
Preterm birth	4951 (15 RCTs)	⊕⊕◯◯ Low^1^^,^^2^	RR 0.79 (0.62, 1.02)	70 per 1000	15 fewer per 1000 (27 fewer to 1 more)
Low birthweight	2178 (7 RCTs)	⊕⊕⊕◯ Low^1^^,^^3^	RR 0.53 (0.38, 0.77)	167 per 1000	78 fewer per 1000 (103 fewer to 38 fewer)

Abbreviations: CI, confidence interval; GRADE, Grading of Recommendations Assessment Development and Evaluation; RCT, randomized controlled trial; RR, risk ratio/relative risk.

1 Downgrade 1 level for risk of bias (performance bias) due to blinding difficulties of participants/personnel.

2 Downgrade 1 level for imprecision due to CI spanning 1.

3 Downgrade 1 level for imprecision due to fewer than 10 trials with small sample sizes (majority <100).

## Discussion

### Summary of main results

This systematic review and meta-analysis of 29 RCTs involving over 7300 participants evaluated the impact of dietary interventions before and/or during pregnancy on preterm birth and low birthweight. The findings support the beneficial role of improving diet quality through interventions based on whole dietary patterns before and during pregnancy in reducing the incidence of low birthweight and potentially preterm birth, although the certainty of evidence is low. These effects varied in significance based on intervention duration, timing, and dietary composition.

Our findings suggest that early initiation and sustained duration of dietary interventions during pregnancy may be associated with reduced risk of adverse birth outcomes, particularly low birthweight. We found potential benefits when interventions begin in the first or second trimester and are maintained for 4–7 mo, though the evidence remains limited for interventions in the preconception period, third trimester, and those of very short or extended duration.

Our review found that quality-focused dietary patterns significantly reduced risk of low birthweight. The observed benefits of interventions that improved diet quality, either by providing recommended macronutrient intakes or by enhancing fat quality through the inclusion of foods rich in unsaturated fats (e.g., nuts, seeds, olive oil, and fatty fish), as seen in dietary patterns such as the Mediterranean or DASH diets, demonstrate the potential role of nutrition-based strategies in lowering low birthweight incidence, likely due to improved placental development, nutrient transport, and fetal tissue accretion [6,66]. Importantly, stratified analysis revealed that these beneficial effects of the identified diets were evident in both high-/upper-middle-income settings, where baseline nutritional adequacy is generally higher, and in lower-middle-income settings, where undernutrition is more common and improvements in dietary quality may yield greater marginal benefits. However, only 1 RCT represented lower-middle-income countries, and no data were available for low-income countries, limiting our ability to draw meaningful conclusions for these populations. Moreover, we observed that baseline risk status modified intervention effects, highlighting the potential for early, diet quality-focused dietary pattern interventions to benefit even low-risk populations in reducing the burden of preterm birth and low birthweight, although data were from 1 trial with high risk of bias.

Although the DASH diet emerged as the most effective in reducing both preterm birth and low birthweight, this finding was based on a single trial with very low certainty of evidence and a small event rate [39]. Nonetheless, our findings suggest the need to shift from single-nutrient interventions toward structured, whole-diet approaches that target overall dietary quality and composition in optimizing birth outcomes before and during early pregnancy. These better reflect real-life dietary behaviors and capture the complex interactions among nutrients that single-nutrient approaches may overlook, though some believe nutritional changes during pregnancy may be difficult and too late [67].

Taken together, our findings highlight the potential importance of initiating dietary interventions in the first trimester and sustaining them for 4–7 mo, particularly those that provide recommended macronutrient intakes or emphasize high-quality fats. We therefore recommend that healthcare professionals consider incorporating such dietary patterns into early pregnancy nutritional counseling.

### Findings in the context of existing literature

Our findings align with existing reports that show a positive trend between dietary interventions and lower risk of adverse birth outcomes, specifically preterm birth and low birthweight [68]. Although 2 reviews found strong associations between dietary interventions and a lower risk of preterm birth [[10], [69]], they also reported an unclear or weak association between dietary interventions and low birthweight, potentially due to methodological differences such as combining diet and exercise interventions [69] or relying heavily on observational studies [10]. In contrast, another review found a relationship between dietary intervention and a lower risk of low birthweight, but no effect on preterm birth [70].

Three systematic reviews with similar robustness evidence found that the DASH and Mediterranean-style diets consistently show promising associations with reduced preterm birth risk, aligning with our findings [10,71,72]. Although 1 review reported mixed results for the Mediterranean diet [72], others highlight its potential, particularly for reducing early preterm birth, though this finding stems from a single cohort study [10], and low birthweight risk [71].

Significant gaps remain in the literature regarding the timing, duration, geographic context, and inclusion of high-risk populations in studies of dietary interventions and preterm birth. Most existing reviews have not adequately considered intervention timing; where it has been explored, the focus has been limited to mid-to-late pregnancy, with findings differing from ours, which suggest that interventions initiated in the first and second trimesters are more effective in reducing low birthweight and preterm birth [68]. Similarly, although the duration of dietary interventions has been largely overlooked, the limited evidence available, focused only on stillbirth, highlights the need for broader analysis [70]. Geographic context has also been insufficiently addressed, with prior reviews tending to focus narrowly on either low- and middle-income countries or high- and upper-middle-income countries, rather than comparing across contexts [10,70]. Finally, research involving high-risk populations has been sparse and often fails to report outcomes directly related to preterm birth or low birthweight [69].

Our review was not specifically focused on assigning a “healthy” or “unhealthy” dietary pattern as demonstrated in previous literature. However, the dietary interventions in the included RCTs were hypothesized to improve diet quality and be more beneficial for the participants than the placebo/control/comparator. Our review includes better quality evidence and search methods and determines any potential effects of the timing, duration, baseline risks of the participants, socioeconomic context, diet type, and macronutrient content of specific dietary interventions before and/or during pregnancy on the outcome of preterm birth and low birthweight.

### Possible mechanisms

Improved diet quality, either by aligning macronutrient intakes with recommended levels or by enhancing fat quality before and during pregnancy, may reduce adverse birth outcomes through several key mechanisms, supported by both human and animal studies. First, adequate and balanced macronutrient intake is crucial for supporting placental function, promoting fetal growth and development, and regulating inflammation [73,74]. The reduction in low birthweight incidence is particularly noteworthy, as it underscores the role of maternal nutrition in optimizing fetal growth. Animal models have been particularly valuable in elucidating these mechanisms, demonstrating that maternal nutrient deficiencies can lead to placental insufficiency and altered fetal programming [75]. For instance, rodent studies have shown that deficiencies or imbalances, particularly in protein and energy during pregnancy, impact placental amino acid transport and fetal growth [75]. For women at high risk of adverse birth outcomes, such as those with GDM, GH, obesity, or who are underweight, targeted dietary interventions that provide essential nutrients can play a significant role in mitigating these risk factors [73].

Second, dietary interventions of improved diet quality, such as a European diet rich in animal protein and lipids, may influence the maternal gut microbiome, the community of microorganisms in the digestive tract, which has been linked to pregnancy outcomes [74]. Animal studies have provided causal evidence for this relationship, showing that improved diet-induced changes in the maternal microbiome can directly affect fetal development and birth timing [74,76]. Human studies support these findings, with evidence that altered gut or low diversity in placental microbiomes is associated with an increased risk of preterm birth and low birthweight [74,77]. These animal and human studies collectively highlight how improved diet quality can influence pregnancy outcomes through both direct nutritional effects and indirect microbiome-mediated pathways.

### Strengths and limitations

Our systematic review and meta-analyses have several notable strengths. Our review employed a rigorous search strategy, yielding a large and geographically diverse sample of RCTs and quasi-RCTs from several countries across most continents. This comprehensive approach enhances the generalizability of findings to various cultural contexts, potentially supporting the established link between low/lower-middle-income status and higher preterm birth/low birthweight rates [5]. However, no representation of low-income countries may limit generalizability to this specific context, despite the large and diverse diets within these RCTs.

This review provides the most up-to-date analysis of dietary patterns, diet quality, and their impact on preterm birth and low birthweight. Our review includes several new RCTs not in prior reviews and employed subgroup analysis to explore potential mechanisms linking diet quality and dietary pattern interventions to adverse birth outcomes, particularly in women predisposed to such complications, such as risk of having the primary outcomes (preterm birth and low birthweight). These findings underscore the potential of improved diet quality based on specific dietary patterns in mitigating adverse pregnancy outcomes.

Our study has several limitations. The overall certainty of evidence was low, largely due to heterogeneity in study designs, population characteristics, and intervention types, which limits the generalizability of the findings. These limitations introduced methodological concerns such as high or unclear risk of bias in many studies and imprecision due to small sample sizes. Additionally, adherence to dietary interventions was inconsistently measured across the included RCTs, potentially influencing the observed effects. Including RCTs from lower-middle-income, upper-middle-income, and high-income countries introduces variability in baseline nutritional status. Women with pre-existing morbidities that may impact nutritional status or lower baseline nutritional status may derive greater benefits from dietary interventions. Our subgroup and stratified analyses suggested potential differences, but additional evidence is needed to strengthen these conclusions. No trials were conducted in low-income countries, which limits our ability to draw conclusions for these populations. Furthermore, although this review comprehensively addresses preterm birth and low birthweight as primary outcomes, data on the severity of preterm birth (very and extremely preterm birth) and the severity of low birthweight (very and extremely low birthweight) were lacking. We were unable to conduct a sensitivity analysis for the outcomes preterm birth and low birthweight where significant heterogeneity was observed due to a limited number of RCTs with a general low risk of bias. Notably, only 1 high-quality RCT contributed data to the outcome for low birthweight. This precluded the planned in-depth analysis, further highlighting the need for more rigorous trials with standardized methodologies. The inherent difficulty of blinding participants and personnel in dietary intervention RCTs posed another limitation, leading to an overall low quality of evidence due to performance and detection bias. Most RCTs exhibited a high or unclear risk of bias due to blinding constraints. Double-blinding was often unfeasible as interventions involved specific food items. In some cases, either investigators or participants were blinded, but not both. Additionally, the lack of preregistration and published protocols for some included RCTs complicated the assessment of reporting bias. Compliance measurement methods also varied (e.g., food frequency questionnaires, 24-hr dietary recall, and dietary logs), raising concerns about self-reporting bias, which reduced the overall quality of the RCTs. We identified variations in the reason for dietary intervention implementation across RCTs, underscoring the need for future trials to define specific dietary patterns that can be widely implemented in diverse populations. A significant limitation we encountered was the small sample sizes and the small number of preterm births in the RCT implementing a DASH diet [39], which had a significant effect on the conclusion supporting the implementation of a diet providing the recommended macronutrient intake, as it was one of the 3 studies for that category. Additionally, the majority of the RCTs for low birthweight contained sample sizes of fewer than 100, including the only RCTs implementing DASH and Mediterranean diets [31,39], limiting statistical power and the reliability and generalizability of these findings. The heterogeneity in study designs, populations, and dietary components also limits the generalizability of findings.

To address these limitations, future research should focus on including larger sample sizes, standardizing dietary interventions, intervention aims, gestational age assessments, and participant inclusion criteria. This may help optimize the benefits of dietary interventions, enhance their generalizability, and improve comparability across studies. Additionally, defining clear eligibility criteria, particularly with respect to baseline nutritional status, would help determine which populations benefit most. Future research should identify critical windows for intervention by trimester and clarify the role of diet quality and specific dietary patterns. Finally, studies should assess the severity of preterm birth and low birthweight to better understand the full impact of improving diet quality based on dietary pattern interventions, especially within larger sample sizes and in low-income countries.

This systematic review and meta-analysis provides evidence that dietary interventions during pregnancy may reduce the incidence of low birthweight and potentially preterm birth, based on low certainty of evidence. The effectiveness of these interventions was evident across different dietary strategies, with comprehensive dietary patterns of recommended macronutrient intake or high unsaturated fat diets showing benefits, potentially when started in the first trimester. Future research should priorities standardized methodologies to assess intervention effectiveness across diverse socioeconomic contexts, include larger sample sizes, explore specific dietary composition recommendations tailored to regional needs, and examine impacts on more severe categories of preterm birth and low birthweight.

## Author contributions

The authors’ responsibilities were as follows – CS, AB: conducted research review and extractions; CS: analyzed data in RevMan 5.4, wrote the manuscript draft and primary responsibility for the final content; and all authors: designed research and protocol, interpreted the data and critically reviewed the manuscript for important intellectual content and read and approved the final manuscript.

## Data availability

Data described in the manuscript, code book, and analytic code will be made available upon agreement with the Data Access Committee via researchhub@auckland.ac.nz. Data will be shared with researchers with a sound proposal on reasonable request.

## Funding

The authors reported no funding received for this study.

## Conflict of interest

The authors report no conflicts of interest.
