# Supplementary material for: Dietary Patterns and Diet Quality before and/or during Pregnancy and How These Affect Birth Outcomes: A Systematic Review and Meta-analysis
Source: Adv Nutr. 2025 Aug 12;16(10):100490. doi: 10.1016/j.advnut.2025.100490 (PMC12455102; doi:10.1016/j.advnut.2025.100490)
Supplement: Multimedia component 1 [file mmc1.docx]

**Dietary patterns and diet quality before and/or during pregnancy and how these affect birth outcomes: a systematic review & meta-analysis**

*Cristal Salatas, Anja Bronnert, Robyn Lawrence, Tanith Alexander, Clare Wall, Frank H Bloomfield, and Luling Lin*

**Supplementary Tables and Figures**

[Supplementary Table 1. 2](#_Toc194596114)

[Supplementary Table 2. 8](#_Toc194596115)

[Supplementary Table 3. 13](#_Toc194596116)

[Supplementary Table 4. 14](#_Toc194596117)

[Supplementary Figure 1. 16](#_Toc194596118)

[Supplementary Figure 2. 17](#_Toc194596119)

[Supplementary Figure 3. 18](#_Toc194596120)

[Supplementary Figure 4. 19](#_Toc194596121)

[Supplementary Figure 5. 20](#_Toc194596122)

[Supplementary Figure 6. 21](#_Toc194596124)

[Supplementary Figure 7. 22](#_Toc194596126)

[Supplementary Figure 8. 23](#_Toc194596128)

[Supplementary Figure 9. 24](#_Toc194596130)

[Supplementary Figure 10. 25](#_Toc194596131)

[Supplementary Figure 11. 26](#_Toc194596133)

[Supplementary Figure 12. 27](#_Toc194596135)

[Supplementary Figure 13. 28](#_Toc194596136)

[Supplementary Figure 14. 29](#_Toc194596137)

[Supplementary Figure 15. 30](#_Toc194596138)

[Supplementary Figure 16. 31](#_Toc194596139)

[Supplementary References 32](#_Toc194596140)

**Supplementary Table 1.** Search strategy to find potential relevant articles for inclusion in the meta-analysis of dietary patterns and diet quality before and/or during pregnancy and how it affects birth outcomes (preterm birth and low birth weight).

| **Search terms and databases** |
| --- |
| Unless otherwise stated, search terms are free text terms. Abbreviations: ’$’: stands for any character; ’?’: substitutes one or no character; adj: adjacent (i.e. number of words within range of search term); exp: exploded MeSH; MeSH: medical subject heading (MEDLINE medical index term); pt: publication type; sh: MeSH; tw: text word |
| **MEDLINE (state platform/delete as appropriate: OvidSP/PubMed/other)**  1  exp infant, newborn/ or Intensive Care, Neonatal/ or Intensive Care Units, Neonatal/ or Gestational Age/  2  (babe or babes or baby* or babies or gestational age? or infant? or infantile or infancy or low birth weight or low birthweight or neonat* or neo-nat* or newborn* or new born? or newly born or premature or pre-mature or pre-matures or prematures or prematurity or pre-maturity or preterm or preterms or pre term? or preemie or preemies or premies or premie or VLBW or VLBWI or VLBW-I or VLBWs or LBW or LBWI or LBWs or ELBW or ELBWI or ELBWs or NICU or NICUs).ti,ab,kw,kf.  3  or/1-2  4  obstetric labor, premature/ or premature birth/  5  ((preterm or premature* or "Pre term" or "pre mature*") adj3 (labo?r* or birth*)).mp. [mp=title, book title, abstract, original title, name of substance word, subject heading word, floating sub-heading word, keyword heading word, organism supplementary concept word, protocol supplementary concept word, rare disease supplementary concept word, unique identifier, synonyms, population supplementary concept word, anatomy supplementary concept word]  6  ((diet or "diet quality") adj3 (score or index)).mp. [mp=title, book title, abstract, original title, name of substance word, subject heading word, floating sub-heading word, keyword heading word, organism supplementary concept word, protocol supplementary concept word, rare disease supplementary concept word, unique identifier, synonyms, population supplementary concept word, anatomy supplementary concept word]  7  maternal nutritional physiological phenomena/ or prenatal nutritional physiological phenomena/ or nutritional requirements/ or nutritional status/  8  ("Diet Quality" or "Dietary Patterns" or "Dietary Guidelines" or "Dietary Score").mp. [mp=title, book title, abstract, original title, name of substance word, subject heading word, floating sub-heading word, keyword heading word, organism supplementary concept word, protocol supplementary concept word, rare disease supplementary concept word, unique identifier, synonyms, population supplementary concept word, anatomy supplementary concept word]  9  controlled clinical trial.pt.  10  randomized controlled trial.pt.  11  randomized.ti,ab.  12  placebo.ti,ab.  13  drug therapy.fs.  14  randomly.ti,ab.  15  trial.ti,ab.  16  groups.ti,ab.  17  or/9-16  18  (quasirandom* or quasi-random* or random*).ti,ab,kw,kf.  19  (control* adj2 (group? or trial? or study)).ti,ab,kw,kf.  20  or/18-19  21  exp animals/ not humans/  22  (or/17,20) not 21  23  ("premature delivery" or "preterm birth*" or "preterm delivery" or "gestation* age" or "gestation* time" or "gestation* length" or "gestation* duration" or "birth weight" or "birthweight" or "neonatal weight" or "neonate weight" or "newborn weight" or "weight at birth" or "birth outcome*").mp. [mp=title, book title, abstract, original title, name of substance word, subject heading word, floating sub-heading word, keyword heading word, organism supplementary concept word, protocol supplementary concept word, rare disease supplementary concept word, unique identifier, synonyms, population supplementary concept word, anatomy supplementary concept word]  24  (diet* pattern* or eating pattern* or diet* guideline* or diet* adherence or diet* compliance or nutrition* adherence or nutritional compliance or diet* score* or diet* quality or diet* index* or diet* indices or diet* habit* or mediterranean diet* or mediterranean style diet* or mediterranean type diet* or healthy eating index or new nordic diet* or dash diet* or ketogenic diet* or paleo diet* or low carb* diet* or high carb* diet* or vegan* diet* or vegetarian* diet* or pescatarian diet*).mp. [mp=title, book title, abstract, original title, name of substance word, subject heading word, floating sub-heading word, keyword heading word, organism supplementary concept word, protocol supplementary concept word, rare disease supplementary concept word, unique identifier, synonyms, population supplementary concept word, anatomy supplementary concept word]  25  3 or 4 or 5 or 23  26  6 or 7 or 8 or 24  27  22 and 25 and 26 |
| **EMBASE (state platform/delete as appropriate: OvidSP/other)**  1  newborn/ or prematurity/ or newborn intensive care/ or newborn care/ or gestational age/  2  (babe or babes or baby* or babies or gestational age? or infant? or infantile or infancy or low birth weight or low birthweight or neonat* or neo-nat* or newborn* or new born? or newly born or premature or pre-mature or pre-matures or prematures or prematurity or pre-maturity or preterm or preterms or pre term? or preemie or preemies or premies or premie or VLBW or VLBWI or VLBW-I or VLBWs or LBW or LBWI or LBWs or ELBW or ELBWI or ELBWs or NICU or NICUs).mp. [mp=title, abstract, heading word, drug trade name, original title, device manufacturer, drug manufacturer, device trade name, keyword heading word, floating subheading word, candidate term word]  3  or/1-2  4  obstetric labor, premature/ or premature birth/  5  ((preterm or premature* or "Pre term" or "pre mature*") adj3 (labo?r* or birth*)).mp. [mp=title, abstract, heading word, drug trade name, original title, device manufacturer, drug manufacturer, device trade name, keyword heading word, floating subheading word, candidate term word]  6  ((diet or "diet quality") adj3 (score or index)).mp. [mp=title, abstract, heading word, drug trade name, original title, device manufacturer, drug manufacturer, device trade name, keyword heading word, floating subheading word, candidate term word]  7  maternal nutritional physiological phenomena/ or prenatal nutritional physiological phenomena/ or nutritional requirements/ or nutritional status/  8  ("Diet Quality" or "Dietary Patterns" or "Dietary Guidelines" or "Dietary Score").mp. [mp=title, abstract, heading word, drug trade name, original title, device manufacturer, drug manufacturer, device trade name, keyword heading word, floating subheading word, candidate term word]  9  Randomized controlled trial/ or Controlled clinical study/  10  random$.ti,ab,kw.  11  Randomization/  12  placebo.ti,ab,kw.  13  ((double or single or doubly or singly) adj (blind or blinded or blindly)).ti,ab,kw.  14  double blind procedure/  15  (controlled adj7 (study or design or trial)).ti,ab,kw.  16  parallel group$1.ti,ab.  17  (crossover or cross over).ti,ab.  18  ((assign$ or match or matched or allocation) adj5 (alternate or group$1 or intervention$1 or patient$1 or subject$1 or participant$1)).ti,ab.  19  (open adj label).ti,ab.  20  (quasirandom* or quasi-random* or random*).ti,ab,kw,kf.  21  (control* adj2 group?).ti,ab,kw,kf.  22  or/9-21  23  (exp animals/ or exp invertebrate/ or animal experiment/ or animal model/ or animal tissue/ or animal cell/ or nonhuman/) and (human/ or normal human/ or human cell/)  24  exp animals/ or exp invertebrate/ or animal experiment/ or animal model/ or animal tissue/ or animal cell/ or nonhuman/  25  (diet* pattern* or eating pattern* or diet* guideline* or diet* adherence or diet* compliance or nutrition* adherence or nutritional compliance or diet* score* or diet* quality or diet* index* or diet* indices or diet* habit* or mediterranean diet* or mediterranean style diet* or mediterranean type diet* or healthy eating index or new nordic diet* or dash diet* or ketogenic diet* or paleo diet* or low carb* diet* or high carb* diet* or vegan* diet* or vegetarian* diet* or pescatarian diet*).mp. [mp=title, abstract, heading word, drug trade name, original title, device manufacturer, drug manufacturer, device trade name, keyword heading word, floating subheading word, candidate term word]  26  ("premature delivery" or "preterm birth*" or "preterm delivery" or "gestation* age" or "gestation* time" or "gestation* length" or "gestation* duration" or "birth weight" or "birthweight" or "neonatal weight" or "neonate weight" or "newborn weight" or "weight at birth" or "birth outcome*").mp. [mp=title, abstract, heading word, drug trade name, original title, device manufacturer, drug manufacturer, device trade name, keyword heading word, floating subheading word, candidate term word]  27  3 or 4 or 5 or 26  28  6 or 7 or 8 or 25  29  24 not 23  30  22 not 29  31  27 and 28 and 30 |
| CENTRAL  #1 MeSH descriptor: [Infant, Newborn] explode all trees 20680  #2 MeSH descriptor: [Intensive Care, Neonatal] this term only 377  #3 MeSH descriptor: [Intensive Care Units, Neonatal] this term only 1036  #4 MeSH descriptor: [Gestational Age] this term only 3975  #5 ("babe" or "babes" or baby* or "babies" or "gestational age" or "gestational ages" or infant? or "infantile" or infancy or "low birth weight" OR "low birth weights" or "low birthweight" or "low birthweights" or neonat* or "neo-nate" or “neo-nates” or newborn* or "new born" or “new borns” or “newly born” or "premature" or "pre-mature" or "pre-matures" or prematures or prematurity or "pre-maturity" or "preterm" or "preterms" or "pre term" or “pre terms” or "preemie" or "preemies" or "premies" or "premie" or "VLBW" or "VLBWI" or "VLBW-I" or "VLBWs" or "LBW" or "LBWI" or "LBWs" or "ELBW" or "ELBWI" or "ELBWs" or "NICU" or "NICUs") 112270  #6 ("Diet Quality" or "Dietary Patterns" or "Dietary Guidelines" or "Dietary Score" or "mediterranean diet" or "mediterranean style diet" or "mediterranean type diet" or "Healthy Eating index" or "New Nordic Diet" or "dash diet" or "ketogenic diet" or "paleo diet" or "vegan diet" or "vegetarian diet" or "pescatarian diet") 6260  #7 ("premature delivery" OR "preterm birth" OR "preterm delivery" OR "gestation age" OR "gestation time" OR "gestation length" OR "gestation duration" OR "birth weight" OR "birthweight" OR "neonatal weight" OR "neonate weight" OR "newborn weight" OR "weight at birth" or "birth outcome") 17966  #8 #1 OR #2 OR #3 OR #4 OR #5 OR #7 112907  #9 #8 AND #6 in Trials 398 |
| **Clinical Trials**   'diet* pattern*' OR 'eat* pattern*' OR 'diet* guideline*' OR 'diet* adhere*' OR 'diet* complian*' OR 'nutrition* adhere*' OR 'nutrition* complian*' OR 'diet* score*' OR 'diet* quality*' OR 'diet* index*' OR 'diet* indices' OR 'diet* habit*' \| Interventional Studies \| preterm birth OR low birth weight OR gestational age OR birthweight \| Studies with Female Participants |
| ***Other databases***  **WHO ICTRP:** (preterm birth OR premature birth OR low birth weight) AND (diet pattern OR eat pattern OR diet guideline OR diet score OR diet quality OR diet index OR mediterranean diet OR new nordic diet OR dash diet)  **CT**: (preterm birth OR premature birth OR low birth weight) AND (diet pattern OR eat pattern OR diet guideline OR diet score OR diet quality OR diet index OR mediterranean diet OR new nordic diet OR dash diet)  **ISRCTN**: (preterm birth OR premature birth OR low birth weight) AND (diet pattern OR eat pattern OR diet guideline OR diet score OR diet quality OR diet index OR mediterranean diet OR new nordic diet OR dash diet) |

Searches were performed through September 17^th^, 2023 and updated November 22^nd^, 2024.

**Supplementary Table 2.** Lists of studies excluded via full-text assessment and reasons for exclusions.

| Unable to extract data |
| --- |
| 1. Duran A. Meddiet at early gestation reduces gestational diabetes mellitus (GDM) incidence and adverse gestational and neonatal outcomes. Nutrients. 2017;66:A387. 2. Frith A, Frongillo E, Naved R, Persson LÅ. Timing of prenatal food supplementation influences birth outcomes in women experiencing stress in Bangladesh. FASEB J. 2010 Apr 1;24. 3. Grant SM, Wolever TM, O'Connor DL, Nisenbaum R, Josse RG. Effect of a low glycaemic index diet on blood glucose in women with gestational hyperglycaemia. Diabetes Res Clin Pract. 2011;91(1):15-22. 4. Lee ACC, Abate FW, Mullany LC, Baye E, Berhane YY, Derebe MM, et al. Enhancing nutrition and antenatal infection treatment (ENAT) study: protocol of a pragmatic clinical effectiveness study to improve birth outcomes in Ethiopia. BMJ Paediatr Open. 2022;6(1):e001327. 5. Napoli A, Festa C, Merola G, Bongiovanni M, Mattei L, Colatrella A. Low glycaemic index and hypocaloric diet therapy versus conventional approach in gestational diabetes/one abnormal value in pregnancy, after medical nutritional therapy failure. Diabetologia. 2010;53(SUPPL. 1):S434–5. |
| Wrong comparator |
| 1. Kizirian NV, Kong Y, Muirhead R, Brodie S, Garnett SP, Petocz P, et al. Effects of a low–glycemic index diet during pregnancy on offspring growth, body composition, and vascular health: a pilot randomized controlled trial. Am J Clin Nutr. 2016 Apr 1;103(4):1073–82. 2. Rhodes ET, Pawlak DB, Takoudes TC, Ebbeling CB, Feldman HA, Lovesky MM, et al. Effects of a low–glycemic load diet in overweight and obese pregnant women: a pilot randomized controlled trial. Am J Clin Nutr. 2010 Dec;92(6):1306–15. 3. Goletzke J, De Haene J, Stotland NE, Murphy EJ, Perez-Rodriguez M, King JC. Effect of a low-glycemic load diet intervention on maternal and pregnancy outcomes in obese pregnant women. Nutrients. 2021 Feb 26;13(3):748. 4. Perichart-Perera O., Balas-Nakash M., Rodriguez-Cano A., Legorreta-Legorreta J., Parra-Covarrubias A., Vadillo-Ortega F. Low glycemic index carbohydrates versus all types of carbohydrates for treating diabetes in pregnancy: a randomized clinical trial to evaluate the effect of glycemic control. Int J Endocrinol. 2012;2012:296017. |
| Wrong intervention |
| 1. Ashorn P, Alho L, Ashorn U, Cheung YB, Dewey KG, Gondwe A, et al. Supplementation of maternal diets during pregnancy and for 6 months postpartum and infant diets thereafter with small-quantity lipid-based nutrient supplements does not promote child growth by 18 months of age in rural Malawi: a randomized controlled trial. J Nutr. 2015;145(6):1345–53. 2. Charkamyani F., Khedmat L., Hosseinkhani A. Decreasing the main maternal and fetal complications in women undergoing in vitro fertilization (IVF) trained by nutrition and healthy eating practices during pregnancy. J Matern Fetal Neonatal Med. 2021;34(12):1855–67. 3. Dodd JM, McPhee AJ, Turnbull D, Yelland LN, Deussen AR, Grivell RM, et al. The effects of antenatal dietary and lifestyle advice for women who are overweight or obese on neonatal health outcomes: the LIMIT randomised trial. BMC Med. 2014;12(1):163. 4. Godfrey KM, Cutfield W, Chan SY, Baker PN, Chong YS, Aris IBM, et al. Nutritional intervention preconception and during pregnancy to maintain healthy glucose metabolism and offspring health (“NiPPeR”): study protocol for a randomised controlled trial. Trials. 2017;18(1):131. 5. Hambidge KM, Krebs NF, Westcott JE, Garces A, Goudar SS, Kodkany BS, et al. Preconception maternal nutrition: a multi-site randomized controlled trial. BMC Pregnancy Childbirth. 2014;14(100967799):111. 6. Hauner H, Vollhardt C, Schneider KTM, Zimmermann A, Schuster T, Amann-Gassner U. The impact of nutritional fatty acids during pregnancy and lactation on early human adipose tissue development. rationale and design of the INFAT study. Ann Nutr Metab. 2009;54(2):97–103. 7. Huynh DTT, Tran NT, Nguyen LT, Berde Y, Low YL, Tey SL. Maternal nutritional adequacy and gestational weight gain in Vietnamese pregnant women. Ann Nutr Metab. 2017;71(Supplement 2):629. 8. Jahan K, Roy SK, Mihrshahi S, Sultana N, Khatoon S, Roy H, et al. Short-term nutrition education reduces low birthweight and improves pregnancy outcomes among urban poor women in Bangladesh. Food Nutr Bull. 2014;35(4):414–21. 9. Kafatos AG, Vlachonikolis IG, Codrington CA. Nutrition during pregnancy: the effects of an educational intervention program in Greece. Am J Clin Nutr. 1989;50(5):970–9. 10. Kusinski LC, Murphy HR, De Lucia Rolfe E, Rennie KL, Oude Griep LM, Hughes D, et al. Dietary intervention in pregnant women with gestational diabetes; protocol for the digest randomised controlled trial. Nutrients. 2020;12(4):1165. 11. Okesene-Gafa KAM, Li M, McKinlay CJD, Taylor RS, Rush EC, Wall CR, et al. Effect of antenatal dietary interventions in maternal obesity on pregnancy weight-gain and birthweight: healthy mums and babies (HUMBA) randomized trial. Am J Obstet Gynecol. 2019;221(2):152.e1-152.e13. 12. Peaceman AM, Kwasny MJ, Gernhofer N, Vincent E, Josefson JL. MOMFIT: a randomized clinical trial of an intervention to prevent excess gestational weight gain in overweight and obese women. Am J Obstet Gynecol. 2017;216(1 Supplement 1):S2–3(abstr). 13. Persson LA, Arifeen S, Ekstrom EC, Rasmussen KM, Frongillo EA, Yunus M. Effects of prenatal micronutrient and early food supplementation on maternal hemoglobin, birth weight, and infant mortality among children in Bangladesh: the MINIMat randomized trial. JAMA. 2012;307(19):2050–9. 14. Ziyenda Katenga-Kaunda L, Iversen PO, Holmboe-Ottesen G, Fjeld H, Mdala I, Kamudoni PR. Dietary intake and processes of behaviour change in a nutrition education intervention for pregnant women in rural Malawi: a cluster-randomised controlled trial. Public Health Nutr. 2020 Sep;23(13):2345-2354. 15. Ashorn P, Alho L, Ashorn U, Cheung YB, Dewey KG, Harjunmaa U, et al. The impact of lipid-based nutrient supplement provision to pregnant women on newborn size in rural Malawi: a randomized controlled trial. Am J Clin Nutr. 2015 Feb;101(2):387–97. 16. Courville AB, Harel O, Lammi-Keefe CJ. Consumption of a DHA-containing functional food during pregnancy is associated with lower infant ponderal index and cord plasma insulin concentration. Br J Nutr. 2011;106(2):208–12. 17. de Kok B, Toe LC, Hanley-Cook G, Argaw A, Ouédraogo M, Compaoré A, et al. Prenatal fortified balanced energy-protein supplementation and birth outcomes in rural Burkina Faso: a randomized controlled efficacy trial. PLoS Med. 2022;19(5):e1004002. 18. Hambidge KM, Westcott JE, Garces A, Figueroa L, Goudar SS, Dhaded SM, et al. A multicountry randomized controlled trial of comprehensive maternal nutrition supplementation initiated before conception: the women first trial. Am J Clin Nutr. 2019;109(2):457–69. 19. Huybregts L, Roberfroid D, Lanou H, Menten J, Meda N, Van Camp J, et al. Prenatal food supplementation fortified with multiple micronutrients increases birth length: a randomized controlled trial in rural Burkina Faso. Am J Clin Nutr. 2009;90(6):1593–600. 20. Janmohamed A, Karakochuk CD, Boungnasiri S, Chapman GE, Janssen PA, Brant R, et al. Prenatal supplementation with corn soya blend plus reduces the risk of maternal anemia in late gestation and lowers the rate of preterm birth but does not significantly improve maternal weight gain and birth anthropometric measurements in rural Cambodian. Am J Clin Nutr. 2016;103(2):559–66. 21. Markhus MW, Hysing M, Midtbo LK, Nerhus I, Naess S, Aakre I, et al. Effects of two weekly servings of cod for 16 weeks in pregnancy on maternal iodine status and infant neurodevelopment: mommy’s food, a randomized-controlled trial. Thyroid. 2021;31(2):288–98. 22. Mridha MK, Matias SL, Chaparro CM, Paul RR, Hussain S, Vosti SA, et al. Lipid-based nutrient supplements for pregnant women reduce newborn stunting in a cluster-randomized controlled effectiveness trial in Bangladesh. Am J Clin Nutr. 2016;103(1):236–49. 23. Nga HT, Quyen PN, Chaffee BW, Anh NT, Ngu T, King JC. Effect of a nutrient-rich, food-based supplement given to rural Vietnamese mothers prior to and/or during pregnancy on birth outcomes: a randomized controlled trial. PLoS One. 2020;15(5):e0232197. 24. Potdar R, Sahariah S, Gandhi M, Kehoe S, Brown N, Sane H, et al. Improving women’s diet quality preconceptionally and during gestation: effects on birth weight and prevalence of low birth weight--a randomized controlled efficacy trial in India (Mumbai maternal nutrition project). Am J Clin Nutr. 2014 Nov;100(5):1257‐68. 25. Radhika MS, Bhaskaram P, Balakrishna N, Ramalakshmi BA. Red palm oil supplementation: a feasible diet-based approach to improve the vitamin A status of pregnant women and their infants. Food Nutr Bull. 2003 Jun;24(2):208–17. 26. Adu-Afarwuah S, Lartey A, Okronipa H, Ashorn P, Zeilani M, Peerson JM, et al. Lipid-based nutrient supplement increases the birth size of infants of primiparous women in Ghana. Am J Clin Nutr. 2015;101(4):835–46. 27. Dwarkanath P, Hsu JW, Tang GJ, Anand P, Thomas T, Thomas A, et al. Energy and protein supplementation does not affect protein and amino acid kinetics or pregnancy outcomes in underweight Indian women. J Nutr. 2016;146(2):218–26. 28. Mantaring J, Benyacoub J, Destura R, Pecquet S, Vidal K, Volger S, et al. Effect of maternal supplement beverage with and without probiotics during pregnancy and lactation on maternal and infant health: a randomized controlled trial in the Philippines. BMC Pregnancy Childbirth. 2018;18(1):193. 29. Stevens B, Watt K, Brimbecombe J, Clough A, Judd JA, Lindsay D. A village-matched evaluation of providing a local supplemental food during pregnancy in rural Bangladesh: a preliminary study. BMC Pregnancy Childbirth. 2018;18(1):286. 30. Magohe A, Mackenzie T, Kimario J, Lukmanji Z, Hendricks K, Koethe J, et al. Pre-and post-natal macronutrient supplementation for HIV-positive women in Tanzania: effects on infant birth-weight and HIV transmission. PLoS One. 2018;13(10):e0201038. 31. Matthews LG, Smyser CD, Cherkerzian S, Alexopoulos D, Kenley J, Tuuli MG, et al. Maternal pomegranate juice intake and brain structure and function in infants with intrauterine growth restriction: a randomized controlled pilot study. PLoS One. 2019;14(8):e0219596. 32. Khan GN, Ariff S, Kureishy S, Sajid M, Rizvi A, Garzon C, et al. Effectiveness of wheat soya blend supplementation during pregnancy and lactation on pregnancy outcomes and nutritional status of their infants at 6 months of age in Thatta and Sujawal districts of Sindh, Pakistan: a cluster randomized-controlled trial. Eur J Nutr. 2021;60(2):781–9. 33. Basu A, Feng D, Planinic P, Ebersole JL, Lyons TJ, Alexander JM. Dietary blueberry and soluble fiber supplementation reduces risk of gestational diabetes in women with obesity in a randomized controlled trial. J of Nutr. 2021 May 1;151(5):1128–38. 34. Callaghan-Gillespie M, Schaffner AA, Garcia P, Fry J, Eckert R, Malek S, et al. Trial of ready-to-use supplemental food and corn-soy blend in pregnant Malawian women with moderate malnutrition: a randomized controlled clinical trial. Am J Clin Nutr. 2017;106(4):1062–9. 35. Ganap EP, Amalia RR, Sugmana PA, Hidayati LI, Hakimi M. The effect of snakehead fish (Channa striata) cookies supplementation on fetal growth and birth outcomes: a randomized clinical trial. Mediterr J Nutr Metab. 2022;15(3):393–406. 36. Li YF, Hu NS, Tian XB, Li L, Wang SM, Xu XB, et al. Effect of daily milk supplementation on serum and umbilical cord blood folic acid concentrations in pregnant Han and Mongolian women and birth characteristics in China. Asia Pac J Clin Nutr. 2014;23(4):567–74. 37. Rossary A, Farges MC, Lamas B, Miles EA, Noakes PS, Kremmyda LS, et al. Increased consumption of salmon during pregnancy partly prevents the decline of some plasma essential amino acid concentrations in pregnant women. Clin Nutr. 2014;33(2):267–73. 38. Zhang DY, Cheng DC, Cao YN, Su Y, Chen L, Liu WY, et al. The effect of dietary fiber supplement on prevention of gestational diabetes mellitus in women with pre-pregnancy overweight/obesity: a randomized controlled trial. Front Pharmacol. 2022;13:922015. 39. Erchick DJ, Lama TP, Khatry SK, Katz J, Mullany LC, Zavala E, et al. Supplementation with fortified balanced energy–protein during pregnancy and lactation and its effects on birth outcomes and infant growth in southern Nepal: protocol of a 2×2 factorial randomised trial. BMJ Paediatr Open. 2023 Nov 1;7(1):e002229. 40. Herman A, Hand LK, Gajewski B, Krase K, Sullivan DK, Goetz J, et al. A high fiber diet intervention during pregnancy: the SPROUT (single goal in pregnancy to optimize outcomes) protocol paper. Contemp Clin Trials. 2024 Feb 1;137:107420. 41. Zavala E, Mohan D, Ali H, Siddiqua TJ, Haque R, Ayesha K, et al. Targeting strategies for balanced energy and protein (BEP) supplementation in pregnancy: study protocol for the TARGET-BEP cluster-randomized controlled trial in rural Bangladesh. Trials. 2024 May 13;25(1):315. 42. Wang D, Shifraw T, Costa JC, Abdelmenan S, Tsegaye S, Berhane Y, et al. Targeting strategies of antenatal balanced energy and protein supplementation in Addis Ababa, Ethiopia: study protocol for a randomized effectiveness study. Trials. 2024 Apr 30;25(1):291. 43. NCT05694520. A pragmatic randomized controlled trial of health impact of pistachios on women with gestational diabetes mellitus. 2023. Internet: https://www.cochranelibrary.com/central/doi/10.1002/central/CN-02517883/full (accessed 2024 April 29). 44. Palmer DJ, Keelan J, Garssen J, Simmer K, Jenmalm MC, Srinivasjois R, et al. Study protocol for a randomised controlled trial investigating the effects of maternal prebiotic fibre dietary supplementation from mid-pregnancy to six months’ post-partum on child allergic disease outcomes. Nutrients. 2022;14(13):2753. 45. Fahmida U. Egg Intervention During Pregnancy in Indonesia. 2021. Internet: https://clinicaltrials.gov/study/NCT04694235 (accessed 2024 April 29). |
| Wrong study design |
| 1. Assaf-Balut C, García de la Torre N, Fuentes M, Durán A, Bordiú E, Del Valle L, et al. A high adherence to six food targets of the Mediterranean diet in the late first trimester is associated with a reduction in the risk of materno-foetal outcomes: the St. Carlos gestational diabetes mellitus prevention study. Nutrients. 2018;11(1):66. 2. Assaf-Balut C, Garcia de la Torre N, Duran A, Fuentes M, Bordiu E, Del Valle L, et al. Medical nutrition therapy for gestational diabetes mellitus based on Mediterranean Diet principles: a subanalysis of the St Carlos GDM Prevention Study. BMJ Open Diabetes Res Care. 2018;6(1):e000550. 3. Donnelly JM, Walsh JM, Byrne J, Molloy EJ, McAuliffe FM. Impact of maternal diet on neonatal anthropometry: a randomized controlled trial. Pediatr Obes. 2015;10(1):52–6. 4. Huybregts L, Roberfroid D, Lanou H, Meda N, Taes Y, Valea I, et al. Prenatal lipid-based nutrient supplements increase cord leptin concentration in pregnant women from rural Burkina Faso. J Nutr. 2013;143(5):576–83. 5. ISRCTN84389045. REduction in the incidence of GEstational DIAbetes mellitus (GDM) with MEDDiet/Lifestyle. 2013. Internet: https://www.cochranelibrary.com/central/doi/10.1002/central/CN-01812230/full (accessed 2024 April 29). 6. Melero V, de la Torre NG, Assaf-Balut C, Jimenez I, Del Valle L, Duran A, et al. Effect of a mediterranean diet-based nutritional intervention on the risk of developing gestational diabetes mellitus and other maternal-fetal adverse events in hispanic women residents in spain. Nutrients. 2020;12(11):1–14. 7. Rasmussen KM, Habicht JP. Maternal supplementation differentially affects the mother and newborn. J Nutr. 2010;140(2):402–6. 8. Stewart CP, Oaks BM, Laugero KD, Ashorn U, Harjunmaa U, Kumwenda C, et al. Maternal cortisol and stress are associated with birth outcomes, but are not affected by lipid-based nutrient supplements during pregnancy: an analysis of data from a randomized controlled trial in rural Malawi. BMC Pregnancy Childbirth. 2015;15(100967799):346. |
| Wrong outcomes |
| 1. Goletzke J, Nga HT, Quyen PN, Ngu T, King JC. Effect of a nutrient-rich, food-based supplement given to rural Vietnamese mothers prior to or during pregnancy on the trajectories of nutrient biomarkers. Nutrients. 2020;12(10):2913. 2. Jorgensen JM, Ashorn P, Ashorn U, Baldiviez LM, Gondwe A, Maleta K, et al. Effects of lipid-based nutrient supplements or multiple micronutrient supplements compared with iron and folic acid supplements during pregnancy on maternal haemoglobin and iron status. Matern Child Nutr. 2018;14(4):e12640. |
| Study terminated/ceased early |
| 1. ISRCTN16896947. Incidence of gestational diabetes mellitus (GDM) and the Mediterranean diet. 2016. Internet: https://www.isrctn.com/ISRCTN16896947?q=1.ISRCTN16896947&filters=&sort=&offset=1&totalResults=1&page=1&pageSize=10 (accessed 2024 April 29). 2. Karlsson T, Augustin H, Lindqvist M, Otten J, Petersson K, Storck-Lindholm E, et al. Effect of the new Nordic diet compared with usual care on glucose control in gestational diabetes mellitus: study protocol for the randomized controlled trial intervention with new Nordic diet in women with gestational diabetes mellitus (iNDIGO). Contemp Clin Trials. 2022 Apr;115:106706. |

**Supplementary Table 3.** Duration of each dietary intervention for the included studies (RCTs).

| **Duration of each dietary intervention** | **n of studies** |
| --- | --- |
| **<1 month** | **0** |
| **1-3 months** | **10** |
| DASH diet | 3 |
| High carbohydrate, low fat (CHOICE) diet | 1 |
| Intervention group 1: Carbohydrate counting diet (CHO); Intervention group 2: Carbohydrate counting (CHO) & DASH diet | 1 |
| Low carbohydrate diet (CHO) | 1 |
| Low glycemic index diet (LGI) | 2 |
| Modestly lower carbohydrate diet (MLC) | 1 |
| Very low energy diets (VLED) | 1 |
| **4-7 months** | **20** |
| Balanced plate nutrition education | 1 |
| DASH diet | 2 |
| Dietary counseling with intention of increasing the intake of unsaturated FA and reducing that of saturated FA | 1 |
| Gestational diabetes mellitus (GDM) Diet | 1 |
| High fiber diet | 1 |
| High-carbohydrate, high-fiber diet (HCF) | 1 |
| High-protein low glycemic index (HPLGI) | 1 |
| Intervention 1: Folic acid and milk (FA+milk); Intervention 2: Milk | 1 |
| Low glycemic index diet (LGI) | 4 |
| Low glycemic index diet (LGI) / Low carb diet | 1 |
| Low Trans Fatty Acid diet | 1 |
| Low-Na diet | 1 |
| Mediterranean-style diet | 3 |
| Prenatal nutrition to control gestational weight gain | 1 |
| **8-9 months** | **0** |
| **>9 months** | **0** |

**Abbreviations**: ADA: American Diabetes Association; BEP: Balanced energy protein; BW: birth weight; CHO: carbohydrate; DASH: dietary approaches to stop hypertension; GA: gestational age; GD/GDM: gestational diabetes mellitus; GH: gestational hypertension; GI: glycaemic index; LBW: low birth-weight; MMN: multiple micronutrient; PTB: preterm birth; RCT, randomised controlled trial; TEI: total energy intake

**Supplementary Table 4.** Participant risk type and the corresponding dietary patterns/focused nutrients implemented for the included studies (RCTs).

| **Risk type and the corresponding dietary patterns/focused nutrients implemented** | **n of studies** |
| --- | --- |
| **100% GDM** | **9** |
| DASH diet | 2 |
| High-carbohydrate, high-fiber diet (HCF) | 1 |
| High carbohydrate, low fat (CHOICE diet) | 1 |
| Intervention group 1: Carbohydrate counting diet (CHO); Intervention group 2: Carbohydrate counting (CHO) & DASH diet | 1 |
| Low carbohydrate diet (CHO) | 1 |
| Low glycemic index diet (LGI) | 2 |
| Modestly lower carbohydrate diet (MLC) | 1 |
| **100% obese + GDM in some participants** | **2** |
| DASH diet | 1 |
| High-protein low glycemic index (HPLGI) | 1 |
| **100% obese + GH and GDM in some participants** | **2** |
| Gestational diabetes mellitus (GDM) Diet | 1 |
| Very low energy diets (VLED) | 1 |
| **100% overweight + GDM in some participants** | **1** |
| Low glycemic index diet (LGI) / Low carb diet | 1 |
| **GDM in some participants** | **5** |
| High fiber diet | 1 |
| Low glycemic index diet (LGI) | 3 |
| Prenatal nutrition to control gestational weight gain | 1 |
| **GH and/or GDM in some participants** | **3** |
| DASH diet | 1 |
| Mediterranean-style diet | 2 |
| **Previous PTB in some participants** | **1** |
| Mediterranean-style diet | 1 |
| **100% diabetic (type 1 and type 2)** | **1** |
| DASH diet | 1 |
| **No/Low Risk** | **6** |
| Balanced plate nutrition education | 1 |
| Dietary counseling with intention of increasing the intake of unsaturated FA and reducing that of saturated FA | 1 |
| Intervention 1: Folic acid and milk (FA+milk); Intervention 2: Milk | 1 |
| Low glycemic index diet (LGI) | 1 |
| Low Trans Fatty Acid diet | 1 |
| Low sodium diet | 1 |

**Abbreviations**: ADA: American Diabetes Association; BEP: Balanced energy protein; BW: birth weight; CHO: carbohydrate; DASH: dietary approaches to stop hypertension; GA: gestational age; GD/GDM: gestational diabetes mellitus; GH: gestational hypertension; GI: glycaemic index; LBW: low birth-weight; MMN: multiple micronutrient; PTB: preterm birth; RCT, randomised controlled trial; TEI: total energy intake

**Supplementary Figure 1.** Subgroup analysis for the duration of the intervention (preterm birth outcome). Results from randomised or quasi-randomised controlled trials. The diamond characterises the overall effect estimate. Data are presented as a risk ratio with 95% CIs, using the random-effects model. CI, confidence interval; df, degrees of freedom; M-H, Mantel-Haenszel.

**
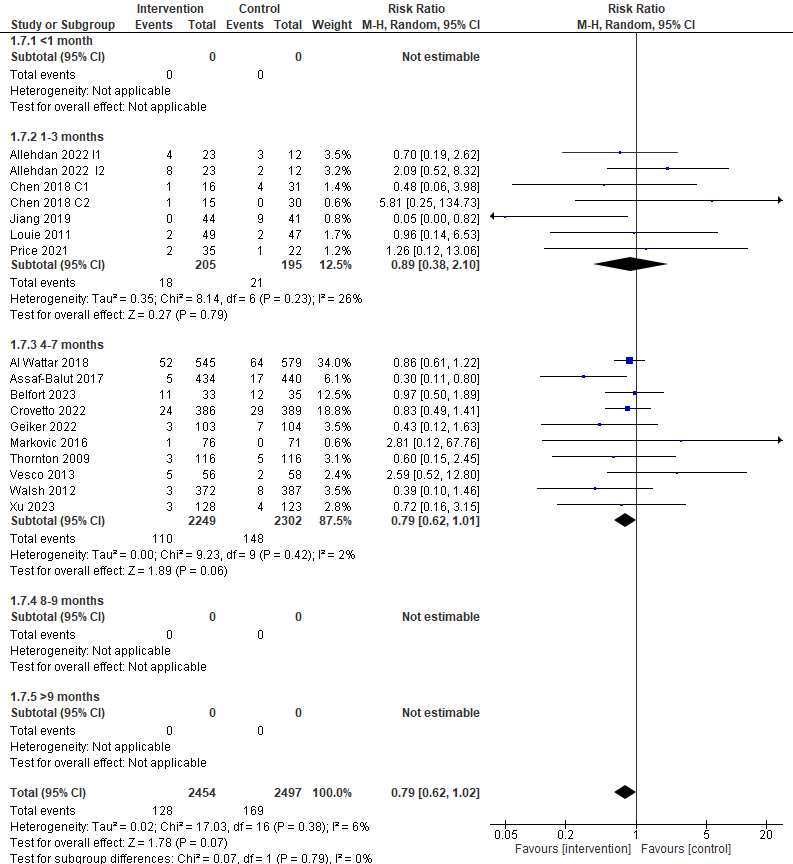
**

**Supplementary Figure 2.** Subgroup analysis for the duration of the intervention (low birth-weight outcome). Results from randomised or quasi-randomised controlled trials. The diamond characterises the overall effect estimate. Data are presented as a risk ratio with 95% CIs, using the random-effects model. CI, confidence interval; df, degrees of freedom; M-H, Mantel-Haenszel.

**
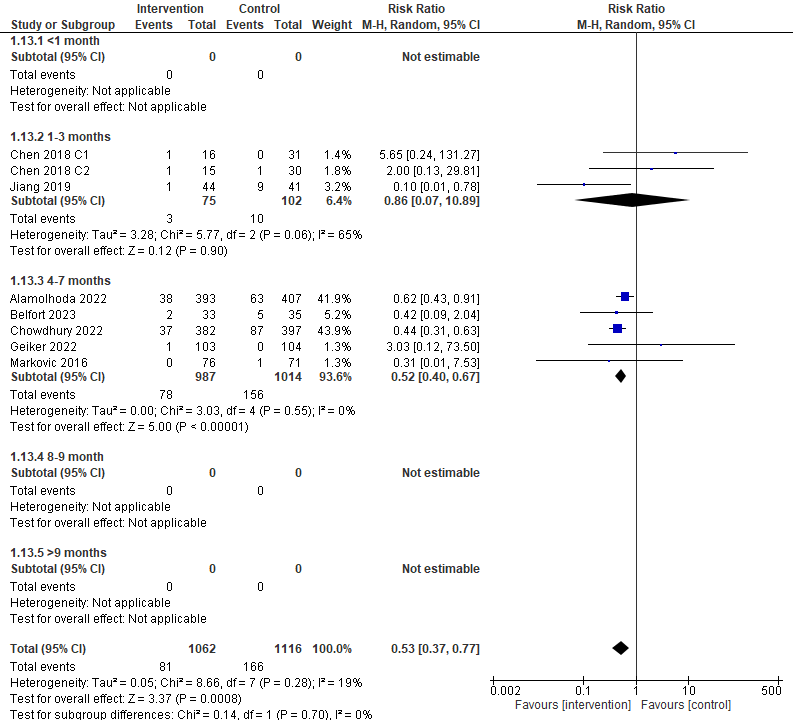
**

**Supplementary Figure 3.** Subgroup analysis for risk (preterm birth outcome). Results from randomised or quasi-randomised controlled trials that reported risk (not all studies reported this). The diamond characterises the overall effect estimate. Data are presented as a risk ratio with 95% CIs, using the random-effects model. CI, confidence interval; df, degrees of freedom; M-H, Mantel-Haenszel.

**
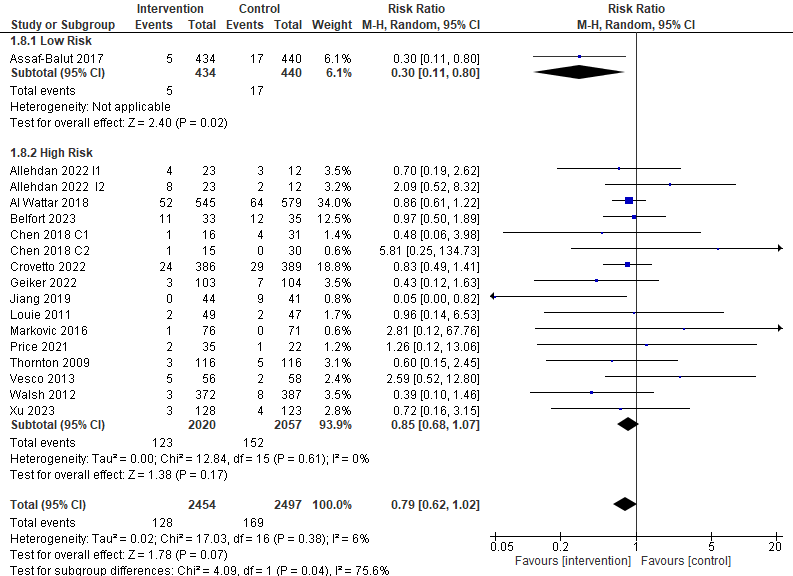
**

**Supplementary Figure 4.** Subgroup analysis for risk (low birth-weight outcome). Results from randomised or quasi-randomised controlled trials that reported risk (not all studies reported this). The diamond characterises the overall effect estimate. Data are presented as a risk ratio with 95% CIs, using the random-effects model. CI, confidence interval; df, degrees of freedom; M-H, Mantel-Haenszel.

**
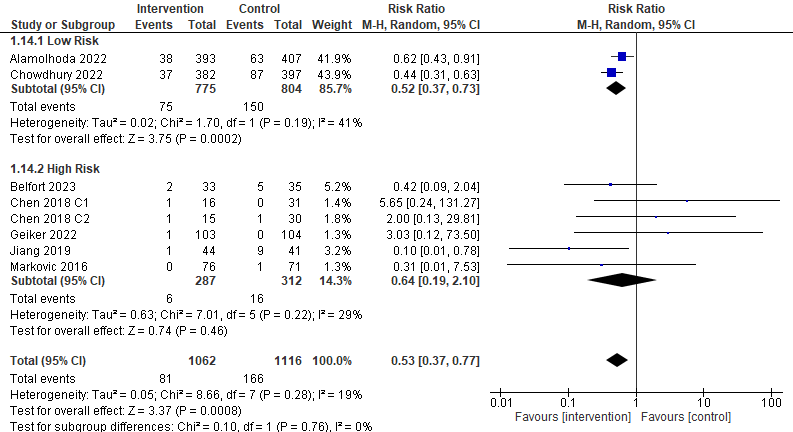
**

**Supplementary Figure 5.** Subgroup analysis for the timing of the intervention (preterm birth outcome). Results from randomised or quasi-randomised controlled trials. The diamond characterises the overall effect estimate. Data are presented as a risk ratio with 95% CIs, using the random-effects model. CI, confidence interval; df, degrees of freedom; M-H, Mantel-Haenszel.

**
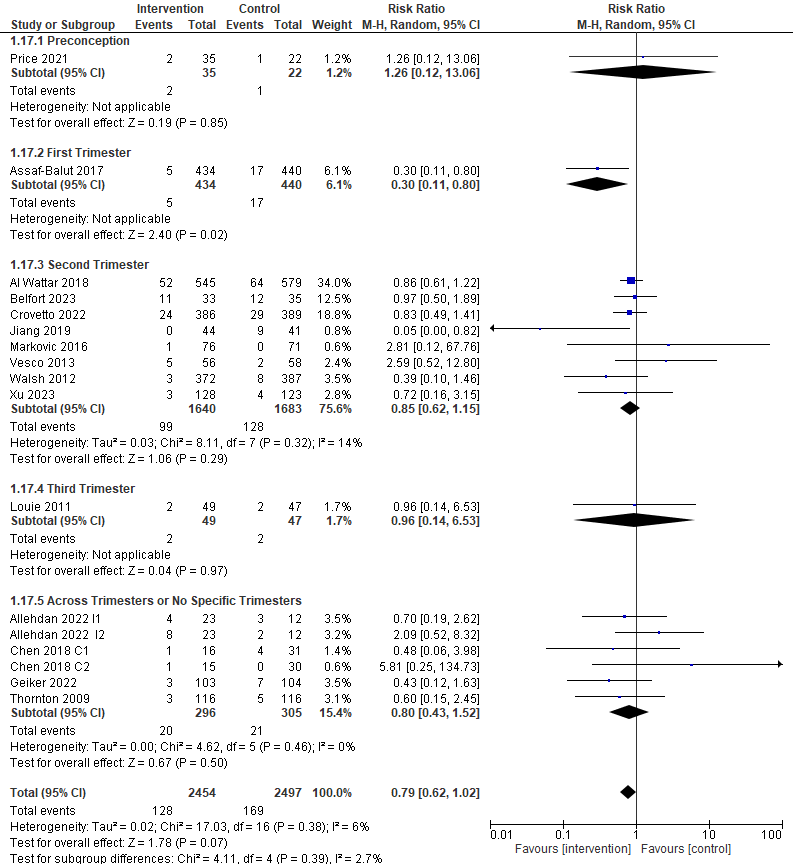
**

**Supplementary Figure 6.** Subgroup analysis for the timing of the intervention (low birth-weight outcome). Results from randomised or quasi-randomised controlled trials. The diamond characterises the overall effect estimate. Data are presented as a risk ratio with 95% CIs, using the random-effects model. CI, confidence interval; df, degrees of freedom; M-H, Mantel-Haenszel.

**
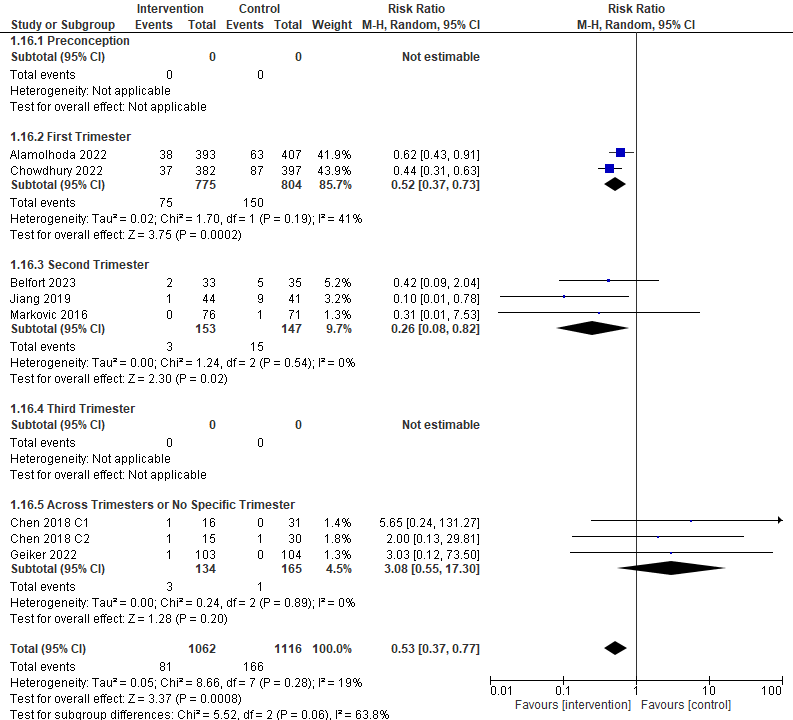
**

**Supplementary Figure 7.** Subgroup analysis for the type dietary patterns implemented as interventions (preterm birth outcome). Results from randomised or quasi-randomised controlled trials. The diamond characterises the overall effect estimate. Data are presented as a risk ratio with 95% CIs, using the random-effects model. CI, confidence interval; df, degrees of freedom; M-H, Mantel-Haenszel.


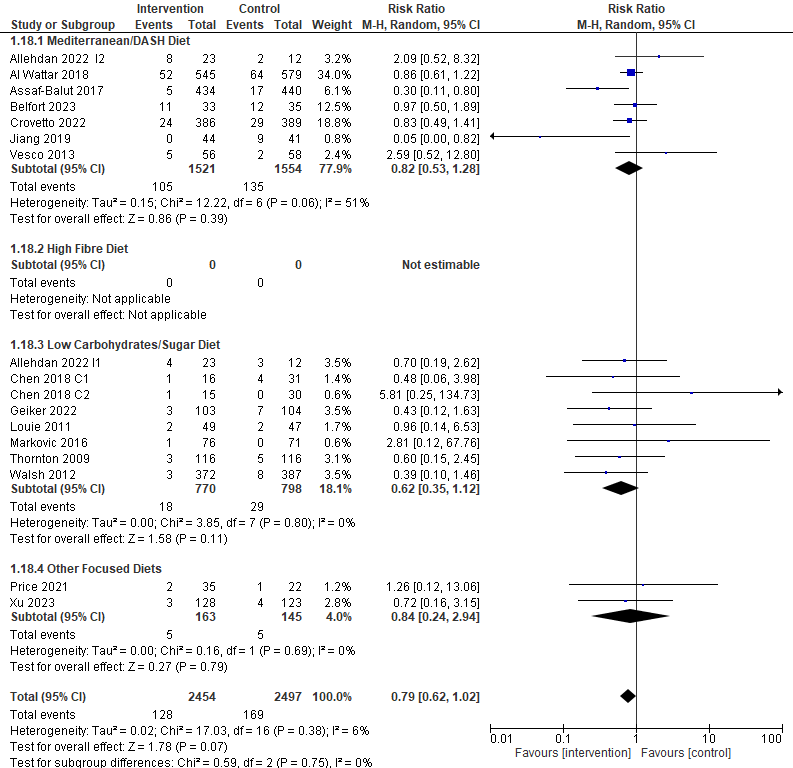


**Supplementary Figure 8.** Subgroup analysis for the dietary patterns implemented as interventions (low birth-weight outcome). Results from randomised or quasi-randomised controlled trials. The diamond characterises the overall effect estimate. Data are presented as a risk ratio with 95% CIs, using the random-effects model. CI, confidence interval; df, degrees of freedom; M-H, Mantel-Haenszel.

**
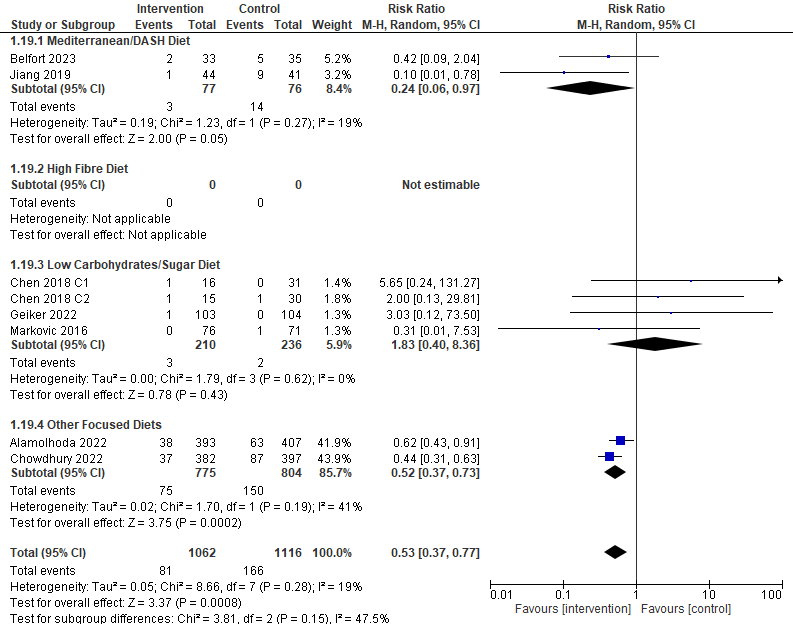
**

**Supplementary Figure 9.** Subgroup analysis for the macronutrient-specific interventions implemented (preterm birth outcome). Results from randomised or quasi-randomised controlled trials that were included in this subgroup analysis. The diamond characterises the overall effect estimate. Data are presented as a risk ratio with 95% CIs, using the random-effects model. CI, confidence interval; df, degrees of freedom; M-H, Mantel-Haenszel.


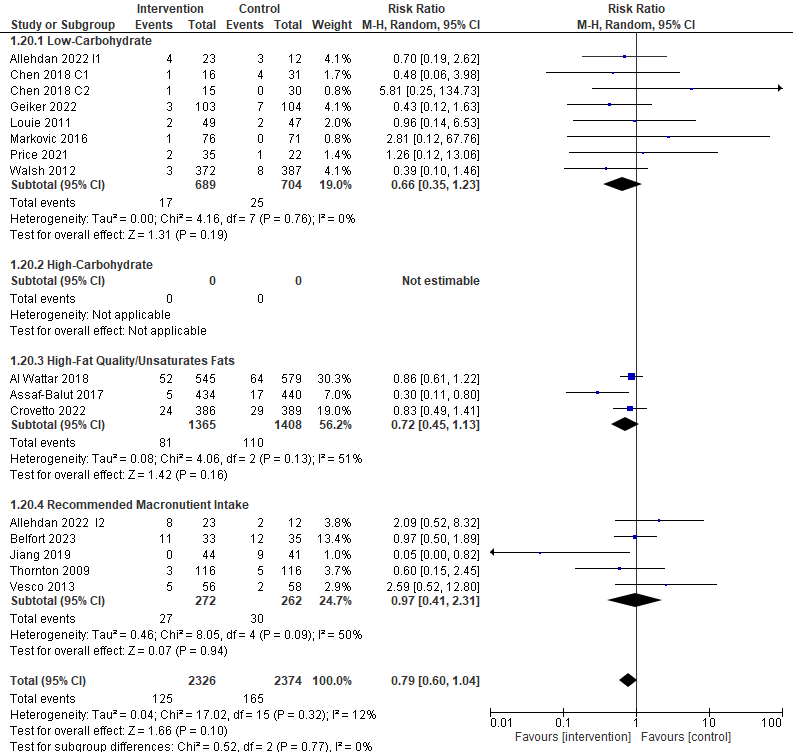


**Supplementary Figure 10.** Subgroup analysis for the study region (preterm birth outcome). Results from randomised or quasi-randomised controlled trials. The diamond characterises the overall effect estimate. Data are presented as a risk ratio with 95% CIs, using the random-effects model. CI, confidence interval; df, degrees of freedom; M-H, Mantel-Haenszel.


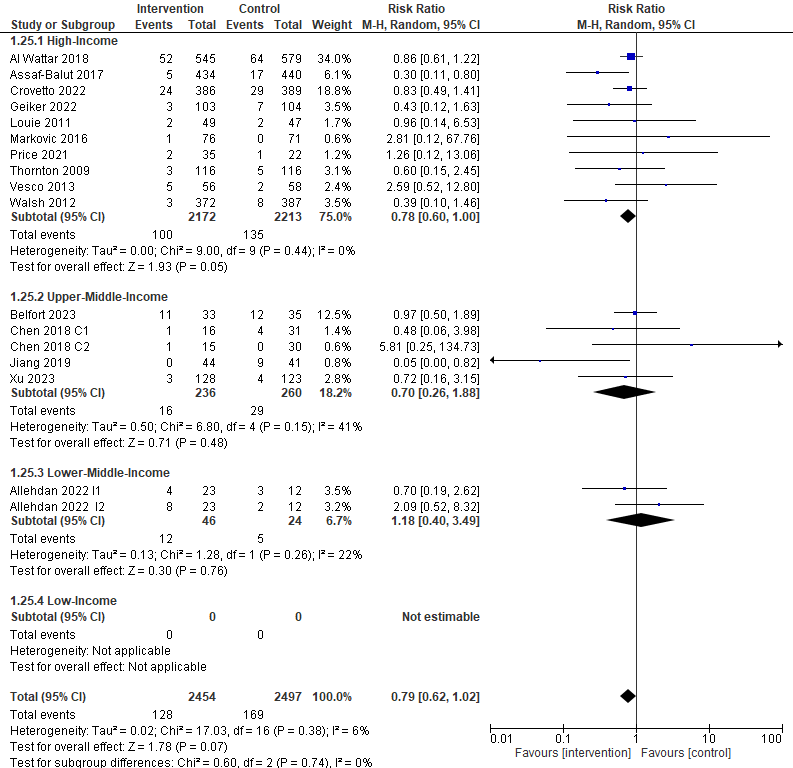


**Supplementary Figure 11.** Subgroup analysis for the study region (low birth-weight outcome). Results from randomised or quasi-randomised controlled trials. The diamond characterises the overall effect estimate. Data are presented as a risk ratio with 95% CIs, using the random-effects model. CI, confidence interval; df, degrees of freedom; M-H, Mantel-Haenszel.

**
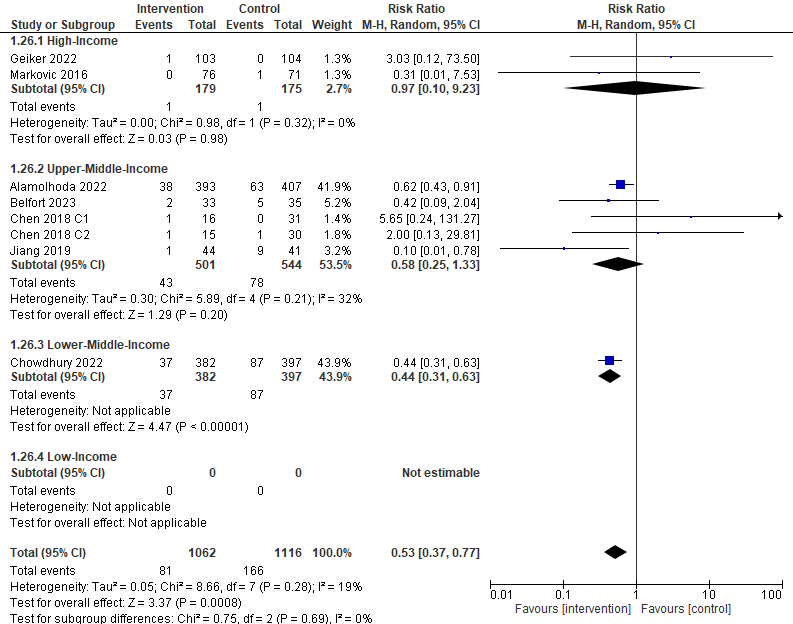
**

**Supplementary Figure 12.** Stratified analyses for the effect of dietary interventions providing the recommended macronutrient intake and high-fat/fat-quality diets on preterm birth in 2 geographical regions. Results from randomised or quasi-randomised controlled trials. The diamond characterises the overall effect estimate. Data are presented as a risk ratio with 95% CIs, using the random-effects model. CI, confidence interval; df, degrees of freedom; M-H, Mantel-Haenszel (47).


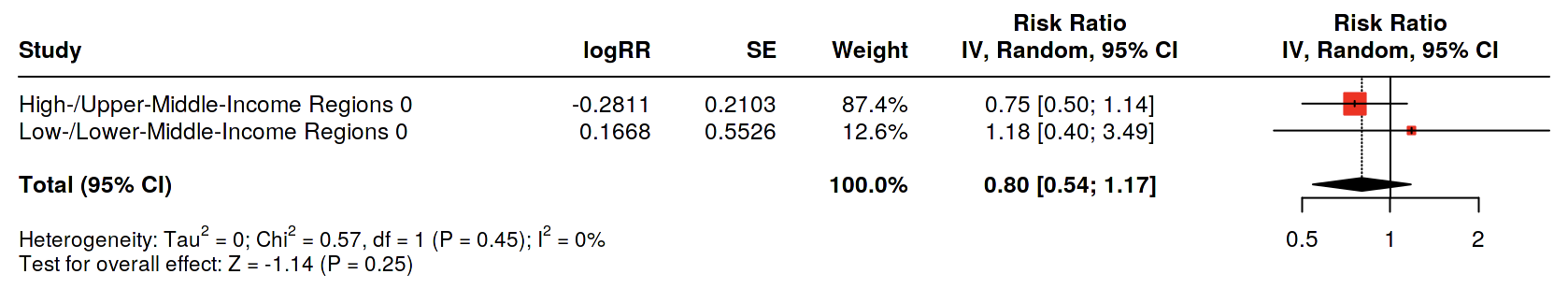

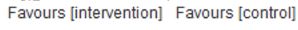


**Supplementary Figure 13.** Stratified analyses for the effect of dietary interventions providing the recommended macronutrient intake and high-fat/fat-quality diets on low birth-weight in 2 geographical regions. Results from randomised or quasi-randomised controlled trials. The diamond characterises the overall effect estimate. Data are presented as a risk ratio with 95% CIs, using the random-effects model. CI, confidence interval; df, degrees of freedom; M-H, Mantel-Haenszel (47).


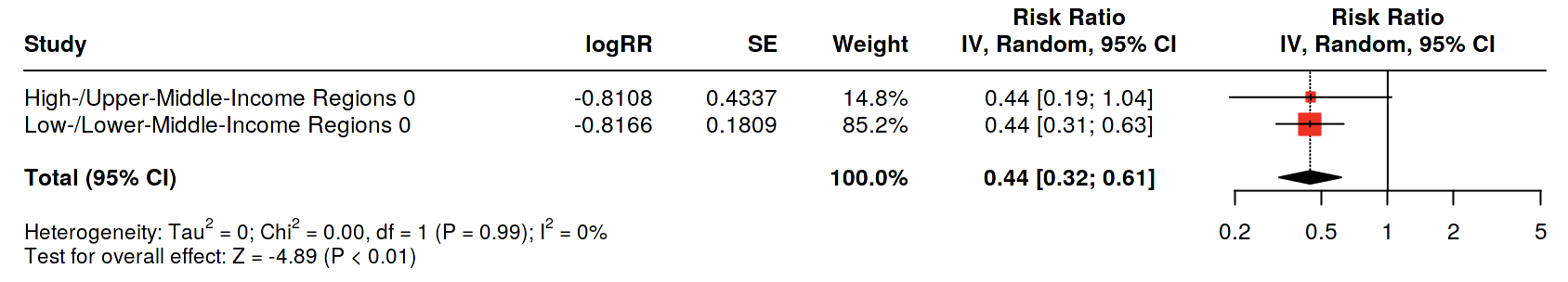

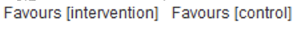


**Supplementary Figure 14.** Funnel plot of the effect of 15 trials on dietary interventions vs comparator and the outcome of preterm birth. The middle vertical line is the overall effect.


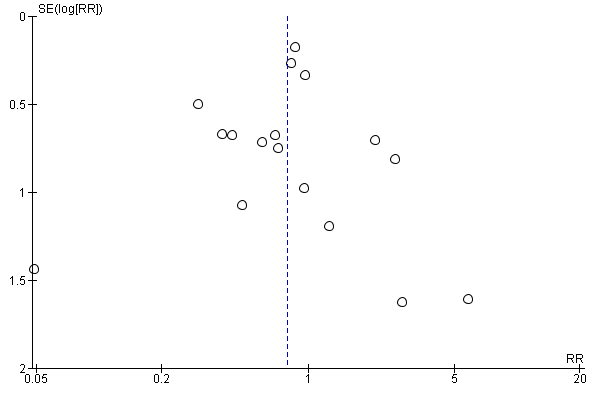


**Supplementary Figure 15.** Funnel plot of the effect of 20 trials on dietary interventions on the outcome of mean gestational age. The middle vertical line is the overall effect.


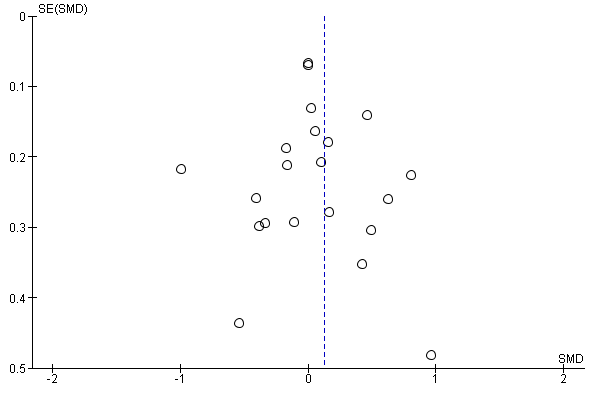


**Supplementary Figure 16.** Funnel plot of the effect of 24 trials on dietary interventions on the outcome of mean birth weight. The middle vertical line is the overall effect.


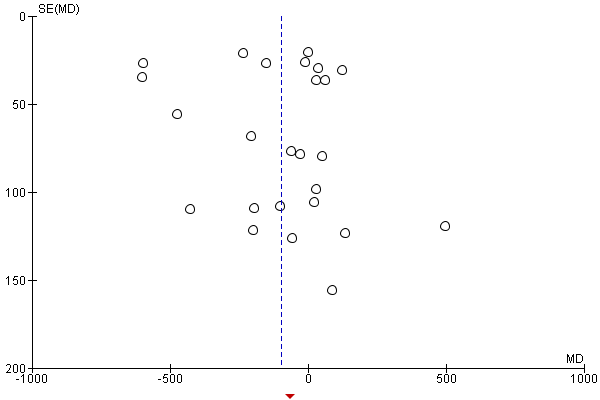


# **Supplementary References**

1. Abreu AM, Young RR, Buchanan A, Lofgren IE, Okronipa HET, Lartey A, et al. Maternal blood pressure in relation to prenatal lipid-based nutrient supplementation and adverse birth outcomes in a Ghanaian cohort: a randomized controlled trial and cohort analysis. J Nutr. 2021;151(6):1637–45.
2. Alamolhoda SH, Asghari G, Mirabi P. Does trans fatty acid affect low birth weight? A randomised controlled trial. J Obstet Gynaecol. 2022 Aug;42(6):2039–45.
3. Allehdan S, Basha A, Hyassat D, Nabhan M, Qasrawi H, Tayyem R. Effectiveness of carbohydrate counting and dietary approach to stop hypertension dietary intervention on managing gestational diabetes mellitus among pregnant women who used metformin: a randomized controlled clinical trial. Clin Nutr. 2022 Feb;41(2):384–95.
4. Al Wattar B. Mediterranean-style diet in pregnancies with metabolic risk factors (esteem): a pragmatic multicente. PLoS Med. 2018;143:192.
5. Al Wattar B, Dodds J, Placzek A, Beresford L, Spyreli E, Moore A, et al. Mediterranean-style diet in pregnant women with metabolic risk factors (ESTEEM): a pragmatic multicentre randomised trial. PLoS Med. 2019 Jul;16(7):e1002857.
6. Al Wattar BH, Dodds J, Placzek A, Spyreli E, Moore A, Hooper R, et al. Effect of simple, targeted diet in pregnant women with metabolic risk factors on maternal and fetal outcomes (ESTEEM): study protocol for a pragmatic multicentre randomised trial. BMJ Open. 2016 Oct 21;6(10):e013495.
7. Asemi Z, Samimi M, Tabassi Z, Esmaillzadeh A. The effect of DASH diet on pregnancy outcomes in gestational diabetes: a randomized controlled clinical trial. Eur J Clin Nutr. 2014 Apr;68(4):490–5.
8. Assaf-Balut C, García de la Torre N, Durán A, Fuentes M, Bordiú E, Del Valle L, et al. A Mediterranean diet with additional extra virgin olive oil and pistachios reduces the incidence of gestational diabetes mellitus (GDM): a randomized controlled trial: the St. Carlos GDM prevention study. PLoS One. 2017;12(10):e0185873.
9. Assaf-Balut C, García de la Torre N, Duran A, Fuentes M, Bordiú E, del Valle L, et al. A Mediterranean diet with an enhanced consumption of extra virgin olive oil and pistachios improves pregnancy outcomes in women without gestational diabetes mellitus: a sub-analysis of the St. Carlos gestational diabetes mellitus prevention study. Ann Nutr Metab. 2018 Dec 14;74(1):69–79.
10. Belfort GP, Farias DR, Padilha P de C, da Silva LBG, dos Santos K, dos Santos MS, et al. Influence of the DASH Diet on gestational weight gain and perinatal outcomes in women with pre-existing diabetes mellitus: a randomized, single-blind, controlled clinical trial. Life. 2023;13(11):2191.
11. Chen H, Liu X, Zou Z, Sun J, Wu J, Xiao M. Effects of low glycemic index cereals on metabolomics and pregnancy outcomes in women with gestational diabetes mellitus. Chinese J Clin Nutr. 2018;26(6):331–7.
12. Chowdhury M, Raynes-Greenow C, Alam A, Dibley MJ. Making a balanced plate for pregnant women to improve birthweight of infants: a study protocol for a cluster randomised controlled trial in rural Bangladesh. BMJ Open. 2017;7(8):e015393.
13. Chowdhury M, Raynes-Greenow C, Kelly P, Alam NA, Afsana K, Billah SM, et al. The impact of antenatal balanced plate nutrition education for pregnant women on birth weight: a cluster randomised controlled trial in rural Bangladesh. Nutrients. 2022;14(21):4687.
14. Crovetto F, Crispi F, Borras R, Paules C, Casas R, Martin-Asuero A, et al. Mediterranean diet, mindfulness-based stress reduction and usual care during pregnancy for reducing fetal growth restriction and adverse perinatal outcomes: IMPACT BCN (improving mothers for a better prenatal care trial Barcelona): a study protocol for a randomized controlled trial. Trials. 2021 May 24;22(1):362.
15. Crovetto F, Crispi F, Casas R, Martin-Asuero A, Borras R, Vieta E, et al. Effects of Mediterranean diet or mindfulness-based stress reduction on prevention of small-for-gestational age birth-weights in newborns born to at-risk pregnant individuals: the IMPACT BCN randomized clinical trial. JAMA. 2021 Dec 7;326(21):2150–60.
16. Dhaded SM, Hambidge KM, Ali SA, Somannavar M, Saleem S, Pasha O, et al. Preconception nutrition intervention improved birth length and reduced stunting and wasting in newborns in South Asia: the women first randomized controlled trial. PLoS One. 2020;15(1):e0218960.
17. Facchinetti F, Vijai V, Petrella E, Gambigliani Zoccoli S, Pignatti L, Di Cerbo L, et al. Food glycemic index changes in overweight/obese pregnant women enrolled in a lifestyle program: a randomized controlled trial. Am J Obstet Gynecol. 2019 Aug;1(3):100030.
18. Fagherazzi S, Farias DR, Belfort GP, dos Santos K, Santana Vieira de Lima T, Silva dos Santos M, et al. Impact of the dietary approaches to stop hypertension (DASH) diet on glycaemic control and consumption of processed and ultraprocessed foods in pregnant women with pre-gestational diabetes mellitus: a randomised clinical trial. Br J Nutr. 2021;126(6):865–76.
19. Geiker NRW, Magkos F, Zingenberg H, Svare J, Chabanova E, Thomsen HS, et al. A high-protein low-glycemic index diet attenuates gestational weight gain in pregnant women with obesity: the “an optimized programming of healthy children” (APPROACH) randomized controlled trial. Am J Clin Nutr. 2022;115(3):970–9.
20. Glosz CM, Schaffner AA, Reaves SK, Manary MJ, Papathakis PC. Effect of nutritional interventions on micronutrient status in pregnant Malawian women with moderate malnutrition: a randomized, controlled trial. Nutrients. 2018;10(7):879.
21. Grant SM, Wolever TMS, O’Connor DL, Nisenbaum R, Josse RG. Effect of a low glycaemic index diet on blood glucose in women with gestational hyperglycaemia. Diabetes Res Clin Pract. 2011;91(1):15–22.
22. H Al Wattar B, Dodds J, Placzek A, Beresford L, Spyreli E, Moore A, et al. Mediterranean-style diet in pregnant women with metabolic risk factors (ESTEEM): a pragmatic multicentre randomised trial. PLoS Med. 2019;16(7):e1002857.
23. Hernandez TL, Farabi SS, Fosdick BK, Hirsch N, Dunn EZ, Rolloff K, et al. Randomization to a provided higher-complex-carbohydrate versus conventional diet in gestational diabetes mellitus results in similar newborn adiposity. Diabetes Care. 2023 Aug 29;46(11):1931–40.
24. Jiang F, Li Y, Xu P, Li J, Chen X, Yu H, et al. The efficacy of the dietary approaches to stop hypertension diet with respect to improving pregnancy outcomes in women with hypertensive disorders. J Hum Nutr Diet. 2019 Dec;32(6):713‐8.
25. Louie JCY, Markovic TP, Perera N, Foote D, Petocz P, Ross GP, et al. A randomized controlled trial investigating the effects of a low-glycemic index diet on pregnancy outcomes in gestational diabetes mellitus. Diabetes Care. 2011;34(11):2341–6.
26. Markovic TP, Muirhead R, Overs S, Ross GP, Louie JCY, Kizirian N, et al. Randomized controlled trial investigating the effects of a low-glycemic index diet on pregnancy outcomes in women at high risk of gestational diabetes mellitus: the GI baby 3 study. Diabetes Care. 2016;39(1):31–8.
27. Mijatovic J, Louie JCY, Buso MEC, Atkinson FS, Ross GP, Markovic TP, et al. Effects of a modestly lower carbohydrate diet in gestational diabetes: a randomized controlled trial. Am J Clin Nutr. 2020;112(2):284–92.
28. Miles EA, Noakes PS, Kremmyda LS, Vlachava M, Diaper ND, Rosenlund G, et al. The salmon in pregnancy study: study design, subject characteristics, maternal fish and marine n-3 fatty acid intake, and marine n-3 fatty acid status in maternal and umbilical cord blood. Am J Clin Nutr. 2011;94(6 Suppl):1986S-1992S.
29. Moreno-Castilla C, Hernandez M, Bergua M, Alvarez MC, Arce MA, Rodriguez K, et al. Low-Carbohydrate diet for the treatment of gestational diabetes mellitus: a randomized controlled trial. Diabetes Care. 2013;36(8):2233–8.
30. Moses RG, Luebcke M, Davis WS, Coleman KJ, Tapsell LC, Petocz P, et al. Effect of a low-glycemic-index diet during pregnancy on obstetric outcomes. Am J Clin Nutr. 2006;84(4):807–12.
31. Ney D, Hollingsworth DR, Cousins L. Decreased insulin requirement and improved control of diabetes in pregnant women given a high-carbohydrate, high-fiber, low-fat diet. Diabetes Care. 1982;5(5):529–33.
32. Niinivirta K, Isolauri E, Laakso P, Linderborg K, Laitinen K. Dietary counseling to improve fat quality during pregnancy alters maternal fat intake and infant essential fatty acid status. J of Nutr. 2011;141(7):1281–5.
33. Niinivirta K, Laakso P, Linderborg K, Poussa T, Isolauri E, Laitinen K. Maternal dietary counseling during pregnancy and infant fatty acid profiles. Int J Food Sci Nutr. 2014;65(3):268–72.
34. Price SAL, Sumithran P, Nankervis AJ, Permezel M, Prendergast LA, Proietto J. Impact of preconception weight loss on fasting glucose and pregnancy outcomes in women with obesity: a randomized trial. Obes Silver Spring Md. 2021;29(9):1445–57.
35. Reece EA, Hagay Z, Gay LJ, O’Connor T, DeGennaro N, Homko CJ, et al. A randomized clinical trial of a fiber-enriched diabetic diet vs. the standard American diabetes association recommended diet in the management of diabetes mellitus in pregnancy. J Mat-fet Inv. 1995;5(1):8–12.
36. Thornton YS, Smarkola C, Kopacz SM, Ishoof SB. Perinatal outcomes in nutritionally monitored obese pregnant women: a randomized clinical trial. J Natl Med Assoc. 2009;101(6):569–77.
37. Toe LC, Bouckaert KP, De Beuf K, Roberfroid D, Meda N, Thas O, et al. Seasonality modifies the effect of a lipid-based nutrient supplement for pregnant rural women on birth length. J Nutr. 2015;145(3):634–9.
38. Urwin HJ, Miles EA, Noakes PS, Kremmyda LS, Vlachava M, Diaper ND, et al. Effect of salmon consumption during pregnancy on maternal and infant faecal microbiota, secretory IgA and calprotectin. Br J Nutr. 2014;111(5):773–84.
39. Vanslambrouck K, de Kok B, Toe LC, De Cock N, Ouedraogo M, Dailey-Chwalibóg T, et al. Effect of balanced energy-protein supplementation during pregnancy and lactation on birth outcomes and infant growth in rural Burkina Faso: study protocol for a randomised controlled trial. BMJ Open. 2021 Mar 24;11(3):e038393.
40. van der Maten GD, van Raaij JM, Visman L, van der Heijden LJ, Oosterbaan HP, de Boer R, et al. Low-sodium diet in pregnancy: effects on blood pressure and maternal nutritional status. Br J Nutr. 1997;77(5):703–20.
41. Vesco KK, Karanja N, King JC, Gillman MW, Leo MC, Perrin N, et al. Efficacy of a group-based dietary intervention for limiting gestational weight gain among obese women: a randomized trial. Obesity (Silver Spring). 2014;22(9):1989–96.
42. Walsh J, Mahony R, Foley M, McAuliffe F. ROLO study: A randomized control trial of low glycemic index diet to prevent macrosomia in euglycemic women. Am J Obstet Gynecol. 2012;206(1 SUPPL. 1):S4.
43. Walsh JM, McGowan CA, Mahony R, Foley ME, McAuliffe FM. Low glycaemic index diet in pregnancy to prevent macrosomia (ROLO study): randomised control trial. BMJ. 2012 Aug 30;345:e5605.
44. Walsh J, Mahony R, Foley M, Mc Auliffe F. A randomised control trial of low glycaemic index carbohydrate diet versus no dietary intervention in the prevention of recurrence of macrosomia. BMC Pregnancy Childbirth. 2010;10(100967799):16.
45. Xu J, Lin X, Fang Y, Cui J, Li Z, Yu F, et al. Lifestyle interventions to prevent adverse pregnancy outcomes in women at high risk for gestational diabetes mellitus: a randomized controlled trial. Front Immunol. 2023;14:1191184.
46. Yao J, Cong L, Zhu B, Wang T. Effect of dietary approaches to stop hypertension diet plan on pregnancy outcome patients with gestational diabetes mellitus. Bangladesh J Pharmacol. 2015;10:732‐38.
47. Fekete JT, Gyorffy B. MetaAnalysisOnline.com: an online tool for the rapid meta-analysis of clinical and epidemiological studies. J Med Internet Res. 2025;27:e64016.
48. McGuinness LA, Higgins JPT. Risk-of-bias VISualization (robvis): an R package and Shiny web app for visualizing risk-of-bias assessments. Res Synth Methods. 2021 Jan;12(1):55-61.
